# Enhancing cell-mediated immunity through dendritic cell activation: the role of Tri-GalNAc-modified PLGA-PEG nanoparticles encapsulating SR717

**DOI:** 10.3389/fimmu.2024.1490003

**Published:** 2024-12-23

**Authors:** Yang Gong, Hongbin Jia, Wenrui Dang, Ting Zhou, Pu He, Xiaolei Wang, Bingdong Zhu

**Affiliations:** ^1^ State Key Laboratory for Animal Disease Control and Prevention & Lanzhou Center for Tuberculosis Research, Institute of Pathogen Biology, School of Basic Medical Sciences, Lanzhou University, Lanzhou, China; ^2^ State Key Laboratory of Applied Organic Chemistry, College of Chemistry and Chemical Engineering, Lanzhou University, Lanzhou, China; ^3^ College of Veterinary Medicine, Lanzhou University, Lanzhou, China

**Keywords:** adjuvant, dendritic cells, Tri-GalNAc, SR717, PLGA-PEG nanoparticles, tuberculosis

## Abstract

**Introduction:**

Vaccines against intracellular pathogens like *Mycobacterium tuberculosis (M. tuberculosis)* require the induction of effective cell-mediated immunity. Adjuvants primarily enhance antigen-induced adaptive immunity by promoting the activation of antigen-presenting cells (APCs).This study is to develop an adjuvant targeted to dendritic cells (DCs), one of the main APCs, so as to assist in inducing a long-term cellular immune response to *M. tuberculosis* protein antigens.

**Methods:**

Polylactic-co-glycolic acid-polyethylene glycol (PLGA-PEG) nanoparticles (NPs) modified with Triantennary N-Acetylgalactosamine (Tri-GalNAc) were prepared to target DCs. Additionally, the stimulator of interferon genes (STING) agonist SR717 was encapsulated within PLGA-PEG NPs to activate DCs. Meanwhile, *M. tuberculosis* fusion protein (TP) was encapsulated in PLGA-PEG NPs to construct vaccine candidates: TP/Tri-GalNAc-PLGA-PEG-SR717 (TP/GPS in short) and TP/ Tri-GalNAc-PLGA-PEG (TP/GP in short). The targeting and activation effects of these NPs were assessed *in vitro* and *in vivo*, and their immunogenicity were evaluated in mice.

**Results:**

Tri-GalNAc modification significantly enhanced the targeting of NPs to DCs, and encapsulated SR717 effectively promoted the maturation and activation of DCs. TP/GPS elicited a potent antigen-specific T cell immune response and successfully induced long-term immune memory in mice. Moreover, after the mice were infected with H37Ra *via* nasal instillation, TP/GPS significantly reduced the bacterial load in their lungs.

**Discussion:**

Tri-GalNAc-modified PLGA-PEG NPs in combination with SR717 targeted and activated DCs, effectively assisting *M. tuberculosis* antigen in inducing long-term T cell-mediated immunity. This approach offers an innovative and effective adjuvant strategy for the development of subunit vaccine against intracellular pathogen.

## Graphical Abstract

*M. Tuberculosis* fusion proteins (TP) and the STING agonist SR717 are co-encapsulated within Triantennary N-Acetylgalactosamine (Tri-GalNAc)-modified PLGA-PEG nanoparticles, denoted as TP/GPS. Following subcutaneous administration, these TP/GPS are effectively phagocytosed by dendritic cells (DCs). The encapsulated TP and SR717 are then released, stimulating the DCs maturation and antigen presentation to T cells. This leads to the subsequent activation and differentiation of CD4^+^ and CD8^+^T cells into memory T cells.

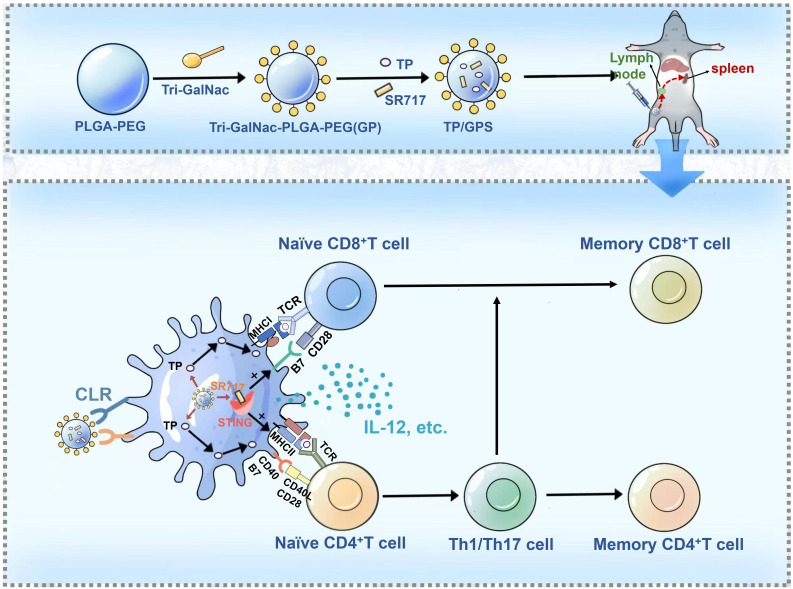

## 1 Introduction

Developing vaccines against intracellular pathogens, such as *Mycobacterium tuberculosis* (*M. tuberculosis*), poses unique challenges due to the pathogens’ ability to reside and replicate within host cells. Effective vaccines must stimulate a robust cell-mediated immune response, especially the T-helper (Th) 1 cells and cytotoxic T lymphocyte (CTLs) to kill the resided pathogen directly or indirectly by activating macrophage (1–4). Furthermore, as tuberculosis (TB) is a chronic disease, an effective TB vaccine should be able to induce the generation of long-term memory T cells (5). After the host is infected with *M. tuberculosis*, long-term memory T cells can rapidly proliferate and differentiate to produce effector cytokines and antimicrobial molecules to kill the pathogen (6, 7). Adjuvants regulate adaptive immune responses by activating innate immunity and assisting in the activation of T and B lymphocytes (8). Selecting appropriate adjuvants to enhance the T cell-mediated immune memory is a promising strategy for TB subunit vaccines (9).

Dendritic cells (DCs) are one of the principal antigen-presenting cells (APCs) that play a crucial role in facilitating adaptive immune responses, especially in generating CD4^+^T and CD8^+^T cell responses (10–13). Hence, the process of directing antigens specifically to DCs can augment antigen presentation and thereby amplify immune activation (14). C-type lectin receptors (CLRs) are phagocytic receptors highly expressed on the surface of immature DCs. These receptors selectively recognize specific carbohydrate structures, facilitating the phagocytosis process of phagocytes (15). CLRs are classified into type I and type II based on the number of conserved carbohydrate-recognition domains (CRDs) (16). Type I CLRs, including the mannose receptor (MR) and CD205, possess multiple CRDs, whereas type II CLRs, such as dendritic cell-specific intercellular adhesion molecule-3-grabbing non-integrin (DC-SIGN) and Macrophage Galactose-Binding Lectin (MGL, CD301), feature a single CRD (17). The MR recognizes antigens with terminal mannose, fucose, glucose, and acetylated glucans (15, 16). Mannosylated phosphatidylethanolamine enhances the binding of liposomes to immature DCs (18, 19). MGL receptor has the capacity to bind to galactose (Gal), N-acetylgalactosamine (GalNAc) and Lewis X (20). Nanoparticles (NPs) modified with GalNAc residues, which could be recognized by MGL receptor (21, 22), can effectively target DCs, accumulate in lymph nodes, and trigger immune responses (23, 24).

Immunostimulants are substances that enhance or modulate the immune response triggered by vaccines by activating the innate immune response (25). Toll-like receptor (TLR) agonists have shown significant adjuvant effects in clinical and animal experiments. They activate specific TLR to initiate signaling pathways, induce the secretion of cytokines and chemokines by DCs, and activate T cells and B cells (26). Additionally, double-stranded DNA molecules (dsDNA) can activate the cyclic GMP-AMP synthase (cGAS)-Stimulator of interferon genes (STING) signaling pathway (27, 28). Upon activation, cGAS can mediate the production of cyclic GMP-AMP (cGAMP), which can be recognized by the STING, inducing the production of IFN-I and promoting the maturation of DCs as well as their migration to the lymph nodes (29, 30). Given the unique contributions of the cGAS-STING pathway to both innate and adaptive immunity, the potential use of agonists of this pathway as vaccine adjuvants has been investigated (31, 32). Studies have shown that immunization of mice with a combination of the *M. tuberculosis* antigen ESAT-6 and the STING agonist c-di-AMP can reduce the bacterial load in the lungs and spleens of the immunized mice (33). Encapsulating the STING agonist cGAMP in liposomal NPs and nasally immunizing mice with the influenza virus H1N1 subtype vaccine can induce tissue-resident memory T (Trm) cells in the lungs (34). Emily N Chin et al. identified a non-nucleotide, small- molecule STING agonist, called SR717, which can specifically bind to the STING (35). Studies have shown that in mouse models, subcutaneous injection of SR717 can promote the activation of CD8^+^ T cells, natural killer cells, and DCs in tumor-associated tissues. Furthermore, SR717 enhances antigen cross-priming, showing promise as an immunomodulator with potential applications in cancer therapy and antiviral strategies (36).

Polylactic acid-co-glycolic acid (PLGA) was a widely used biodegradable and biocompatible polymer material (37). It has been extensively utilized in various biomedical applications for many years and is approved by both the U.S. food and drug administration (FDA) and european medicines agency (EMA) (38). This study modified PLGA-PEG NPs with Triantennary N-Acetylgalactosamine (Tri-GalNAc) to target DCs through interaction with MGL receptor on DCs. Additionally, SR717, a STING agonist, was co-encapsulated with *M. tuberculosis* protein antigens in the PLGA-PEG NPs. The targeting and activation abilities of these NPs on DCs were tested *in vitro*. Following vaccination, immune responses were investigated, with a particular focus on the T-cell immune memory responses.

## 2 Materials and methods

### 2.1 Materials

PLGA (the ratio of lactide: glycolide = 50:50, MW: 40 kDa, Xianruixibio, China). NH_2_-PEG-N_3_, NH2-PEG-Alkyne (MW: 2 kDa, Ponsure biotechnology, China). Alkyne-Mannose (MW: 2 kDa, Xianruixibio, China). Anti-CD11c-PerCP-Cy5.5 (eBioscience, USA), anti-CD40-FITC (eBioscience, USA), anti-CD80-PE (eBioscience, USA), anti-CD4-FITC (Biolegend, USA), anti-CD8-FITC (Biolegend, USA), anti-CD8-PE (Biolegend, USA), anti-IL-2-PE (Biolegend, USA), anti-IFN-γ-APC (Biolegend, USA), anti-IL-17A-Percp-Cy5.5 (Biolegend, USA). IL-12p70 Mouse Enzyme-linked immunosorbent assay (ELISA) Kit (Dakewe Biotech Company Ltd., Shenzhen, China), IL-10 Mouse ELISA Kit (Dakewe Biotech Company Ltd., Shenzhen, China). Ovalbumin (OVA) (Solarbio, China), granzyme B Mouse ELISA Kit (Beyotime Biotechnology, China), 7H10 solid medium (Becton, Dickinson and Company, USA), FITC (Solarbio, Beijing, china), DiI (Solarbio, Beijing, china), DiR (Med Chem Express, MCE), DAPI (Solarbio, Beijing, china).

### 2.2 Animals

six-eight weeks-old female C57BL/6 and Balb/c mice were purchased from the Lanzhou Veterinary Research Institute and housed under specific pathogen-free conditions at Lanzhou University. Animal experiments were conducted following approved from the Institutional Animal Care and Use Committee of Lanzhou University (Approval jcyxy20211202). All experimental procedures were in accordance with the Institutional Animal Care and Use Committee of Lanzhou University.

### 2.3 Bacterial strains

*M. tuberculosis* H37Ra (ATCC25177) and Bacillus Calmette-Guerin (BCG, Danish strain) bacteria were donated by Fudan University and Lanzhou Institute of Biological Products respectively. BCG and H37Ra are first inoculated onto Lowenstein-Jensen medium, and after 7-14 days, they are transferred to Sauton’s medium. After 4 weeks, the cultures are centrifuged at 6000 revolutions per minute (rpm) for 10 minutes to harvest the bacteria. The bacterial strains are ground with a sterile grinder to prepare a dispersed bacterial suspension. The bacterial suspension is resuspended in PBS, mixed with 50% glycerol, and aliquoted into 1.5ml Eppendorf tubes, which are then stored at -80°C. One of the frozen bacterial strains is taken out and used to count the colonies on 7H10 solid medium.

### 2.4 Synthesis of the SR717, PLGA-PEG, Man-PLGA-PEG, and Tri-GalNAc-PLGA-PEG

#### 2.4.1 Synthesis of Tri-GalNAc-N_3_ and SR717

On the one hand, the synthesis of the target product, Tri-GalNAc-N3, can be delineated through a series of well-defined chemical reactions. Initially, 6-bromocaproic acid, a cost-effective starting material, undergoes a classical substitution reaction to yield the azide compound 1-1. Simultaneously, trimethylol aminomethane hydrochloride and acrylonitrile are employed as starting materials in a classical Michael reaction, resulting in the formation of intermediate 1-2. This intermediate is subsequently transformed into triester compound 1-3 via hydrochloric acid hydrolysis. Following this, compound 1-1 is subjected to a condensation reaction with 1-3 under HBTU activation conditions, leading to the formation of compound 1-4. This compound then undergoes a series of hydrolysis and condensation reactions to yield intermediate 1-6. In a parallel sequence, D-glucosamine hydrochloride serves as the starting material to produce intermediates 1-11 through functional group transformations and glycosidation reactions. Finally, a straightforward ammoniation reaction with 1-6 facilitates the conversion of the intermediates into the desired target product, Tri-GalNAc-N3 (**Supplementary Figure S1**) (39).

On the other hand, the synthesis of the SR717 target compound follows a logical sequence of reactions. First, we utilized inexpensive 6-chlorpyridazin-3-methyl formate and imidazole as starting materials in a classical SNAr reaction to produce intermediate 2-1. This intermediate was then condensed with 2-amino-4,5-difluorobenzoate under HATU activation conditions, resulting in the formation of the SR717 precursor compound 2-2. Finally, the target SR717 compound was obtained through saponification hydrolysis using lithium hydroxide (**Supplementary Figure S2**) (35).

#### 2.4.2 Synthesis of PLGA-PEG

**Scheme 1:**
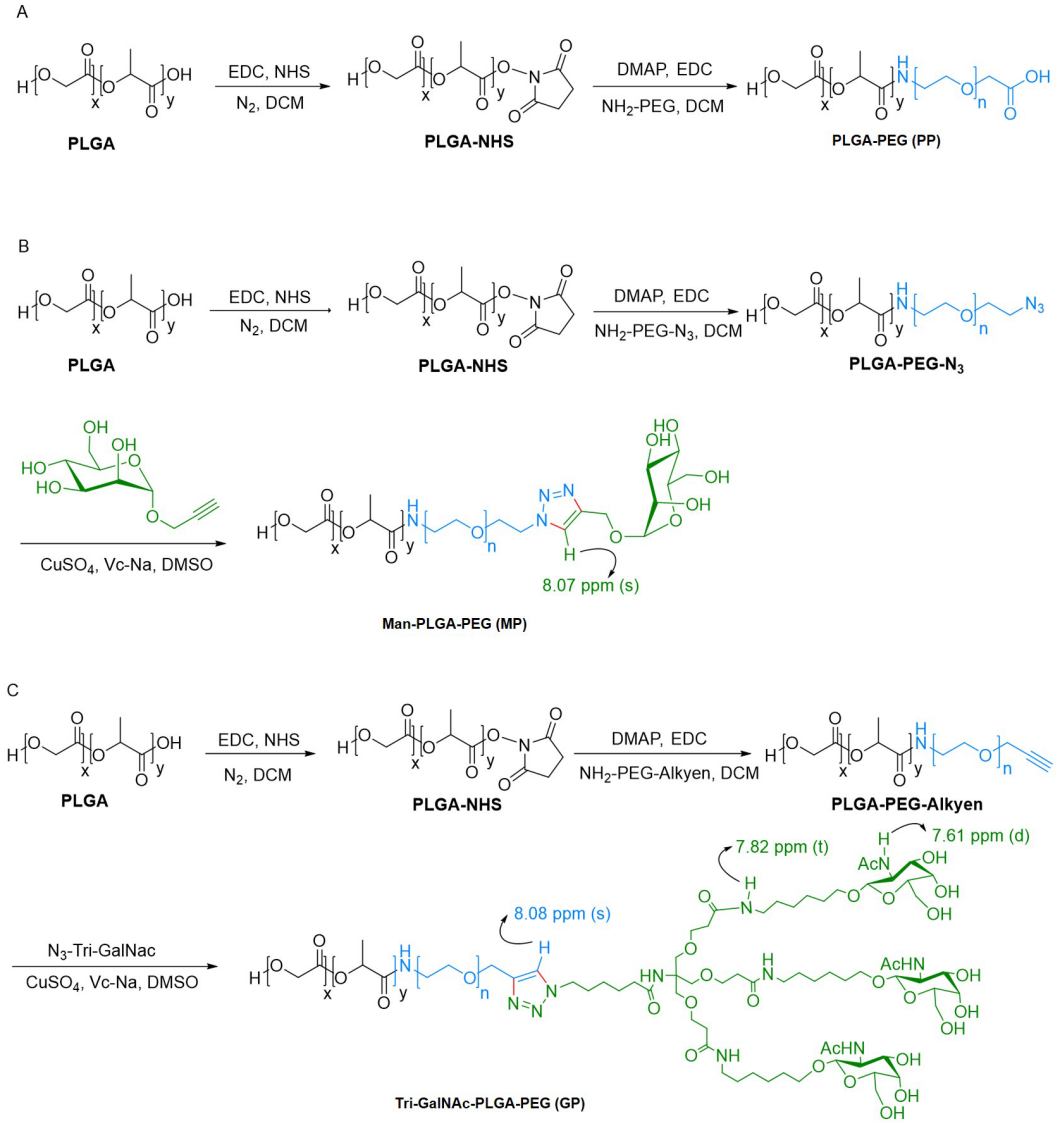
Synthetic route of PP, MP, and GP. **(A)** PP was synthesized via amide reaction. **(B, C)** MP and GP was synthesized through bioorthogonal reaction.

The synthesis of PLGA-PEG (PP) was achieved through an amide reaction by conjugation PLGA-COOH with NH_2_-PEG, adhering to a two-step procedure detailed in prior literature (40, 41). In the first step, PLGA (0.8 g; 0.02 mmol), EDC-HCl (38.34 mg; 0.2 mmol), and N-Hydroxysuccinimide (NHS) (23 mg; 0.2 mmol) were dissolved in dichloromethane (DCM) and stirred for 24 h at room temperature. The solution was subsequently precipitated by the gradual addition of ice-cold ether. The solid precipitate formed was washed three times using a mixture of ice-cold ether and methanol to eliminate any remaining NHS. 12000 rpm, centrifuged for 20 minutes, collected the solid precipitate and thoroughly dried under vacuum to obtain the activated PLGA. In the second step, PEG-NH_2_ (0.102 g; 0.03 mmol) and activated PLGA (0.4 g; 0.01 mmol) were dissolved in DCM. Subsequently, DMAP (6 mg; 0.05 mmol), EDC-HCl (19.17 mg; 0.05 mmol), and 60 μL of triethylamine were added to the reaction mixture. The system was allowed to proceed for 24 h at room temperature. Following this, the reaction mixture was precipitated with cold ether and then washed with a mixture of methanol and ether to remove any unreacted PEG-NH_2_. The precipitated product was collected by centrifugation at 12000 rpm for 20 minutes and then dried under vacuum to yield the final product, PP (**Scheme 1A**).

#### 2.4.3 Synthesis of Man-PLGA-PEG

The synthesis of Man-PLGA-PEG (MP) commenced with the reaction of activated PLGA with NH2-PEG-N3, as outlined earlier, to produce PLGA-PEG-N3. Subsequently, MP was synthesized by conjugation PLGA-PEG-N_3_ with Propargyl α-D-Mannopyranoside through a biorthogonal reaction (42). For this reaction, PLGA-PEG-N_3_ (0.1 g; 0.0025 mmol), propargyl α-D-mannopyranoside (5.4 mg; 0.025 mmol), and Vc-Na (4.95 mg; 0.025 mmol) were dissolved in dimethylsulfoxide (DMSO). The addition of a catalytic amount of CuSO_4_ initiated the reaction, and the mixture was stirred for 2 h at room temperature. Ultrapure water was added dropwise to induce precipitation. The precipitate solid was then washed with ultrapure water to remove any excess propargyl α-D-Mannopyranoside. After centrifugation and vacuum drying of the collected solid, MP was obtained (**Scheme 1B**).

#### 2.4.4 Synthesis of Tri-GalNAc-PLGA-PEG

As previously mentioned, the activated PLGA was reacted with NH_2_-PEG-Alkyen to yield PLGA-PEG-Alkyen. Following this initial reaction, PLGA-PEG-Alkyen was further reacted with N_3_-Tri-GalNAc, resulting in the formation of Tri-GalNAc-PLGA-PEG (GP) (**Scheme 1C**).

### 2.5 Preparation of NPs

OVA or TP and SR717 were encapsulated in PP, MP, or GP using a double emulsion solvent evaporation method to form NPs (41). Briefly, OVA (1 mg) or TP (2mg) was dissolved in 1 mL PBS (aqueous phase), while SR717 (2 mg) and 50 mg PP, MP, or GP were dissolved in 5 mL DCM (organic phase), respectively. Both solutions were sonicated in a ultrasonic processor for 4 min at 500 W in an ice bath using a 2-mm stepped microhead. The secondary emulsion was further emulsified with the aqueous phase containing 2% (w/v) Polyvinyl alcohol (PVA) for 8 min using high pressure homogenizer at 400 bar. The final emulsion was stirred for 4 h to evaporate any DCM. Finally, the NPs were collected by centrifugation at 12,000 × rpm for 20 min and washed three times.

### 2.6 NPs characterization

#### 2.6.1 Particle size and zeta potential analysis

Dilute the NPs with double-distilled water to prepare a 20-fold dilution to achieve a suitable detection range. The particle size and polydispersity coefficient (PDI) of the particles were then assessed using a Brookhaven Nanoparticle Size Analyzer, operating at a measurement angle of 90° and a temperature of 25°C. Following this, the zeta potential of the NPs was also determined using the same Brookhaven Nanoparticle Size Analyzer.

#### 2.6.2 Encapsulation efficiency and loading percentage of antigen and SR717

To calculate the encapsulation rate of the antigen, the protein content in the supernatant was quantified using a bicinchoninic acid (BCA) protein assay kit. The encapsulated protein within the NPs was then calculated by subtracting the protein amount in the supernatant from the total protein present initially. For the detection of SR717 within the NPs, the compound was analyzed using liquid chromatography-mass spectrometry (LC-MS). This analysis was preceded by dissolving the NPs in dimethyl sulfoxide (DMSO) to facilitate the extraction and measurement of SR717.


Encapsulation efficiency (EE)=A/B×100%

Loading percentage (LP)=A/C×100%

In this context, A represents the amount of protein or SR717 in the NPs, B is the total amount of protein or SR717 added, and C denotes the total mass of the NPs.

#### 2.6.3 Scanning electron microscope analysis

Took an appropriate amount of sample and placed them onto the sample column. Allowed it to dry completely. Nest, transferred the sample column to the gold-coating chamber for the gold-coating process. After coating, placed the sample column onto the carrier stage of the JSM-6701F scanning electron microscope (SEM) for observation.

#### 2.6.4 Transmission electron microscope analysis

Took an appropriate amount of sample and placed them on a copper grid coated with a carbon support film, allowing them to air dry naturally. Then, apply an appropriate amount of 1% phosphotungstic acid solution for negative staining. Allow the staining solution to adsorb on the grid for a certain period, remove excess staining solution with filter paper, and ensure the sample is completely dry. Finally, place the dried sample under a 200kV biological cryo-transmission electron microscope (TEM) for observation.

### 2.7 Isolation and culture of bone marrow-derived dendritic cells

Bone marrow-derived dendritic cells (BMDCs) were harvested from the femur and tibia of C57BL/6 mice (6–8 weeks) and lysed using red blood cell lysis buffer, following previously described methods (43). The obtained cells were cultured in six-well culture plates (1 × 10^6^ cells/mL; 5 mL per well) in Roswell Park Memorial Institute (RPMI) 1640 complete medium supplemented with 20 ng/mL recombinant mouse granulocyte-macrophage colony-stimulating factor (GM-CSF), and 10 ng/mL IL-4 at 37°C with 5% CO_2_. On day 2, the entire medium was replaced with fresh RPMI 1640 complete medium that had been supplemented with GM-CSF and IL-4. On day 4, half of the medium was replaced with fresh RPMI 1640 complete medium that had been supplemented with GM-CSF and IL-4. On day 6, non-adherent and loosely adherent cells, identified as immature BMDCs, were harvested for subsequent experiments.

### 2.8 *In vitro* growth inhibition assay of NPs

To determine if the NPs could influence the growth of DCs, we utilized BMDCs. Briefly, in a 96-well plate, 100 µL of the NPs, serially diluted to concentrations ranging from 6.25 µg/mL to 800 µg/mL, were added to BMDCs at a final density of 1×10^4^ cells per well. The plates were incubated for 24 h before the addition of 10 µL of CCK8. After a further 2h incubation, the absorbance was measured at 450 nm using a microplate reader. All experiments were performed with at least three replicate wells per group.

### 2.9 Evaluation of NPs targeting DCs

#### 2.9.1 Assessment the delivery of the NPs *in vivo*

BALB/c mice (6–8 weeks) were injected subcutaneously in the inguinal region with either free 1,1-dioctadecyl-3,3,3,3-tetramethylindotricarbocyaine iodide (DIR) or DIR-labeled NPs (500 ug per mouse). NPs kinetics were examined at 12, 24, 48, 72, 96, and 120 h post-injection by a broad-spectrum small animal *in vivo* optical imaging system with 765-nm excitation and 665-nm emission. Throughout the experiments, mice were anesthetized with 2% isoflurane/O_2_ (v/v) and maintained under 1% isoflurane/O_2_ (v/v). Kinetics was measured by quantifying the fluorescent intensity at the injection site and the inguinal lymph node using DPM fluorescence image video analysis software.

#### 2.9.2 Investigation of the NPs uptake by BMDCs *in vitro*

##### 2.9.2.1 The uptake of NPs by BMDCs *in vitro* was observed using laser confocal scanning microscopy.

BMDCs were seeded at a density of 1 × 10^6^ cells per well in confocal dishes and incubated with fluorescein isothiocyanate (FITC)-labeled NPs at a concentration of 250 µg per well for 3 h. Afterward incubation, the cells were washed twice with PBS and fixed with 4% paraformaldehyde. The cell membranes were labeled with 10 μmol of 1,1-Dioctadecyl-3,3,3,3- tetramethylindocarbocyanine iodide (DiI). Subsequently, the cells were washed twice with PBS, and the nuclei were stained with a 10 μg/mL 4',6-Diamidino-2-Phenylindole (DAPI) solution. The subcellular localization of each target signal was observed using a laser scanning confocal microscope.

##### 2.9.2.2 Flow cytometric analysis of the NPs uptake by BMDCs *in vitro*

BMDCs were seeded at a density of 1 × 10^6^ cells per well in 12-well plates and incubated with FITC-labeled NPs at a concentration of 250 µg per well for 3 h. After incubation, the cells were collected and washed three times with PBS. The uptake of NPs was measured using flow cytometry.

#### 2.9.3 Assessment of NPs targeting to DCs of lymph nodes *in vivo*

Female C57BL/6 mice (6–8 weeks) were immunized subcutaneously with DiI-labeled NPs at a dose of 500 µg per mouse. Mice injected with PBS served as controls. 40 h post-immunization, the inguinal lymph nodes were collected and gently ground to release immune cells. For detecting DCs uptake, the collected immune cells were first stained with anti-CD11c-PerCP-Cy5.5 for 30 min at 4°C, followed by flow cytometry analysis.

### 2.10 Evaluation of NPs’ activation of DCs

#### 2.10.1 Evaluation for the activation and maturation of BMDCs by NPs *in vitro*

Prior to stimulation, induced BMDCs were seeded into 12-well plates at a density of 1×10^6^ cells per well. On the following day, OVA (6 μg/well), SR717 (10 μg/well), LPS (100ng/well) and NPs (OVA content was 6 μg/well, SR717 content was 10 ug/well) were added to co-culture for 24 h, respectively, and three replicate wells were set up in each group. After incubation, the supernatants were collected for detection of cytokines. The presence of secreted IL-12p70 and IL-10 cytokines was quantified using a specific ELISA kit, strictly adhering to the manufacturer’s instructions. For phenotypic analysis, BMDCs were harvested and co-stained with anti-CD11c-PerCP-Cy5.5, anti-CD40-FITC, and anti-CD80-PE antibodies for 30 min at 4°C. Finally, the stained cells were analyzed using flow cytometry.

#### 2.10.2 Evaluation for the activation of DCs by NPs in lymph node *in vivo*

Female C57BL/6 mice (6–8 weeks) were immunized subcutaneously with NPs containing 6 µg of OVA and10 µg of SR717. Mice injected with PBS served as controls. 48 h post-immunization, inguinal lymph node cells were collected, and subsequently stained with anti-CD11c-PerCP-Cy5.5 and anti-CD40-FITC for 30 min at 4°C. The stained cells were then analyzed using flow cytometry.

### 2.11 Evaluation of the immunogenicity and protective efficacy of *M. tuberculosis* fusion protein NPs

#### 2.11.1 Preparation of *M. tuberculosis* fusion protein NPs

*M. tuberculosis* fusion protein LT33 (ESAT6-CFP10-Rv1738) and LT57 (Rv0518-Rv2541) (44) were prepared as previous described (**Supplementary Figure S6**). To prepare the *M. tuberculosis* fusion protein LT33+LT57 (TP) NPs (TP/NPs), we replaced OVA (1mg) with TP (2mg) and added SR717 (20mg), following the procedure outlined in section 2.5. Different types of NPs were produced, including TP/PP (TP/PLGA-PEG), TP/GP (TP/Tri-GalNAc-PLGA-PEG), and TP/GPS (TP/Tri-GalNAc-PLGA-PEG-SR717).

#### 2.11.2 TP/NPs immunization schedule

Mice were divided into six groups: TP, TP/PP, TP/GP, TP/GPS, BCG, and PBS control. The BCG group received an initial injection at week 0, while other groups were immunized at weeks 0, 3, and 6. The BCG group was given 5 × 10^6^ CFU; the TP, TP/PP, TP/GP, and TP/GPS groups received 10 µg of protein per immunization; the TP/GPS group also received 200 µg of SR717 per immunization. Immune responses were measured 6 weeks after the final dose, and immune memory was assessed 12 weeks later. Protective efficacy was evaluated through an intranasal challenge with avirulent *M. tuberculosis* H37Ra (5×10^6^ CFU per mouse) at 12 weeks after the final dose (**Supplementary Figure S7**).

#### 2.11.3 Detection of antigen-specific T cells induced by TP/NPs

Six weeks after the final vaccination, the T cell immune response induced by the TP/NPs was evaluated by detecting the secretion of cytokines from splenic lymphocytes using intracellular cytokine staining (ICS) (45). The specific procedure is as follows: lymphocytes isolated from the spleens were cultured at a density of 5 × 10^6^ cells per well in a 24-well plate. The cells were stimulated with individual antigens of TP (10 μg/mL) for 12 h at 37°C in a 5% CO2 environment. Following stimulation, the cells were blocked by 10% fetal bovine serum (FBS), and stained for surface markers with anti-CD4-FITC. The cells were then permeabilized using the BD Cytofix/Cytoperm kit and stained intracellularly with anti-IL-2-PE and anti-IL-17A-PerCP-Cy5.5, according to the manufacturer’s instructions, and analyzed by flow cytometry. The level of granzyme B secreted by lymphocytes was measured with an ELISA kit.

Memory T cells are categorized into central memory T (T_CM_) cells and effector memory T (T_EM_) cells. T_CM_ cells can survive for several years, while T_EM_ cells are short-lived, typically surviving for 4 to 8 weeks, and provide immediate but temporary protection (46, 47). To observe long-lived memory T cells induced by TP/NPs, T cell-mediated immune responses were analyzed 12 weeks after the final vaccination as previously described (46). Mice were injected subcutaneously with 10 µg of the individual antigens of TP antigen to promote the differentiation of T_CM_-like long-term memory T cells into T_EM_ cells or effector T cells (T_eff_) *in vivo*, 3 days prior to immune detection. After euthanasia, spleen cells were isolated and stimulated *in vitro* with individual antigens of TP antigens (10 µg/m) for 12 h. During this period, T_EM_ cells developed into Teff cells and secreted cytokines. As described in 2.11.3, intracellular cytokine staining was analyzed using flow cytometry, and the level of granzyme B secreted by lymphocytes was measured with an ELISA kit, to indirectly assess the functionality of memory T cells. Flow cytometry gating strategy was shown in **Supplementary Figures S8A, B**.

#### 2.11.4 EdU proliferation assay for memory T cells induced by TP/NPs

5-Ethynyl-2’-deoxyuridine (EdU) is incorporated into the DNA of proliferating T cells and can be detected after memory T cells have undergone proliferation and division. 12 weeks after the final immunization, mice were subcutaneously immunized with 10 µg of individual antigens of TP antigens. 3 days later, splenic lymphocytes were collected and treated with individual antigens of TP antigens and EdU (10 µM) at a density of 5×10^6^ cells per well in a 24-well plate for 3 days. Following the manufacturer’s instructions for the Click-iT™ EdU flow cytometry detection kit, the cells were harvested, fixed, permeabilized, and incubated with Click-iT reaction buffer. Subsequently, the cells were stained with anti-CD4-FITC and anti-CD8-PE antibodies. Flow cytometry was then performed to assess the proliferation capacity of CD4^+^T and CD8^+^T cells. Flow cytometry gating strategy was shown in **Supplementary Figure S8C**.

#### 2.11.5 Detection of specific antibodies in serum of mice

At 6 weeks after the last immunization, antigen-specific immunoglobulin IgG, IgG1, and IgG2c were detected in serum using an ELISA. First, 0.5 µg/well of LT33 or LT57 was added to the plates and incubated overnight at 4°C. The plates were then blocked with 5% skimmed milk powder and incubated with serially diluted serum samples at 37°C for 2 h. After washing, 100 µL of a 1:8000 dilution of goat anti-mouse IgG, rabbit anti-mouse IgG1, and rabbit anti-mouse IgG2c were added to each well. The reaction was developed by adding 100 µL of 3,3′,5,5′-tetramethylbenzidine (TMB) substrate and incubating at room temperature for 10 min. The reaction was then stopped by adding 50 µL of diluted sulfuric acid (1 mol/L) per well. The optical density was quantified at 450 nm. Serum from the PBS group served as the negative control. The antibody titer was determined by identifying the highest serum dilution with optical density value greater than 2.1 times that of the negative control, and the geometric mean titer (GMT) was calculated.

#### 2.11.6 *M. tuberculosis* H37Ra infection and bacteria-load detection

Mice were anesthetized intraperitoneally with 1% sodium pentobarbital at a dose of 50 mg/kg. Each group of mice was infected intranasally with 5 × 10^6^ CFU of H37Ra. 3 weeks after the intranasal infection, the lungs of the infected animals were harvested. The organs were ground and resuspended in PBS, and the resulting dilutions were plated on Middlebrook 7H10 plates containing oleic acid/albumin/dextrose/catalase (OADC). The colony-forming units (CFU) were subsequently counted.

### 2.12 Statistical analysis

Data were evaluated using GraphPad Prism 8.0 software, and the unpaired two-tailed Student′s t-test was used for two-group comparisons, and one-way analysis of variance (ANOVA) was used for multiple group comparisons, followed by Tukey’s *post-hoc* test. The differences were considered statistically significant at *p < 0.05*.

## 3 Results

### 3.1 Characterization of PP, MP, and GP

**Figure 1:**
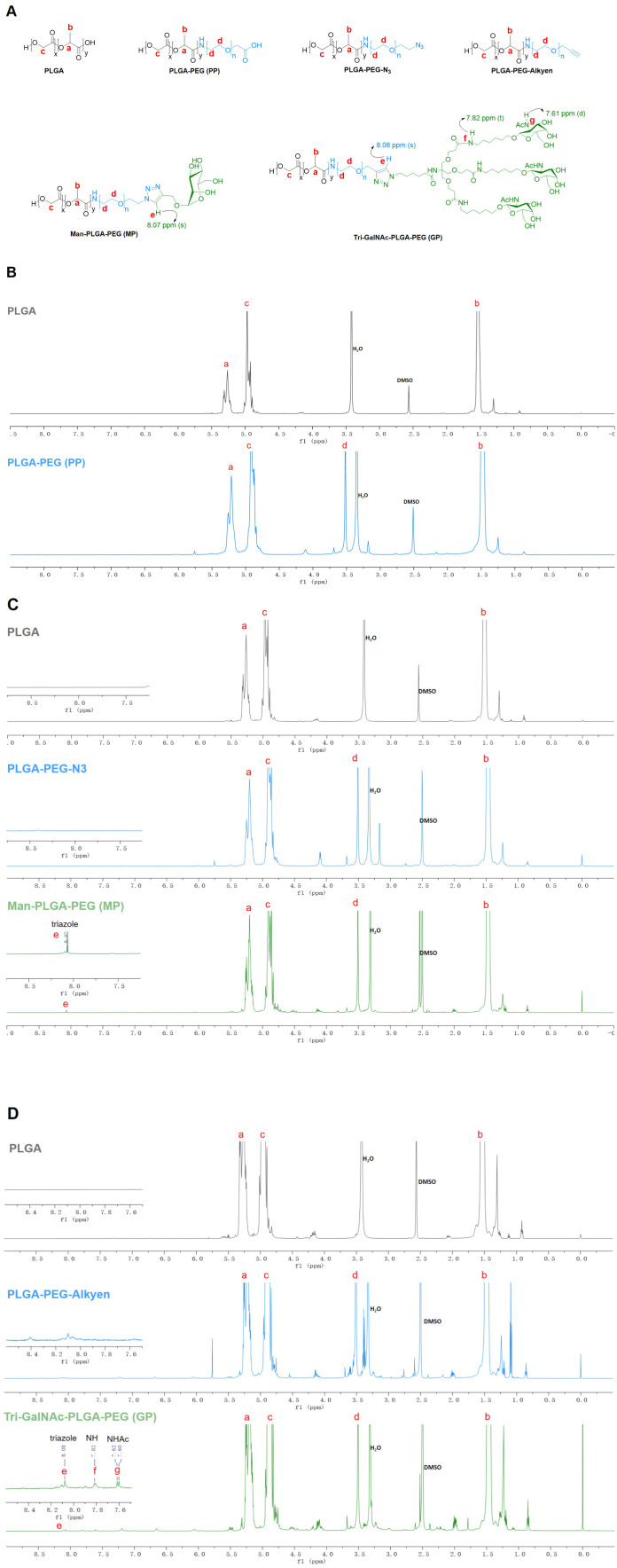
1H NMR spectra of PP, MP, and GP. The PP, MP, and GP were dissolved in DMSO-d6 at a concentration of 10mg/mL, and their chemical composition were characterized using ^1^H NMR spectral analysis. **(A)** Chemical structures of different compounds. **(B)** 1H NMR spectra of PLGA and PLGA-PEG (PP). **(C)** 1H NMR spectra of PLGA, PLGA-PEG-N3, and Man-PLGA-PEG (MP). **(D)** 1H NMR spectra of PLGA, PLGA-PEG-Alkyen, and Tri-GalNAc-PLGA-PEG (GP).

PP was synthesized through an NHS ester coupling reaction, while MP and GP were synthesized *via* bioorthogonal reactions, as illustrated in **Scheme 1**. The chemical structures of PP, MP and GP are shown in **Figure 1A**. Their ^1^H-NMR spectrums are shown in **Figure 1**, with peaks labeled to correspond to specific proton signals according to their molecular structures. For PP, the characteristic peaks include peak a (5.21 ppm, -OCH(CH3) CONH-), peak b (1.48 ppm, -OCH(CH3) CONH-), peak c (4.92 ppm, -OCH2COO-), and peak d (3.52 ppm, -CH2CH2O-) (**Figure 1B**). MP exhibits similar peaks to PP, with the additional peak e (8.07 ppm, -CH-) (**Figure 1C**). The mannose moiety, primarily localized at the polymer’s terminus, results in relatively subdued peak intensities, with characteristic signals ranging from 3.0 to 4.0 ppm. The spectrum of GP extends that of MP, with the additional peak f (7.82 ppm, NH-) and peak g (7.61 ppm, -NHAc-) (**Figure 1D**). In the ^1^H-NMR spectrums, the characteristic peaks of mannose and Tri-GalNAc are overshadowed by those of PP; however, MP and GP were confirmed by a distinct triazole peak at 8.07 ppm.

### 3.2 Characterization of NPs

**Figure 2:**
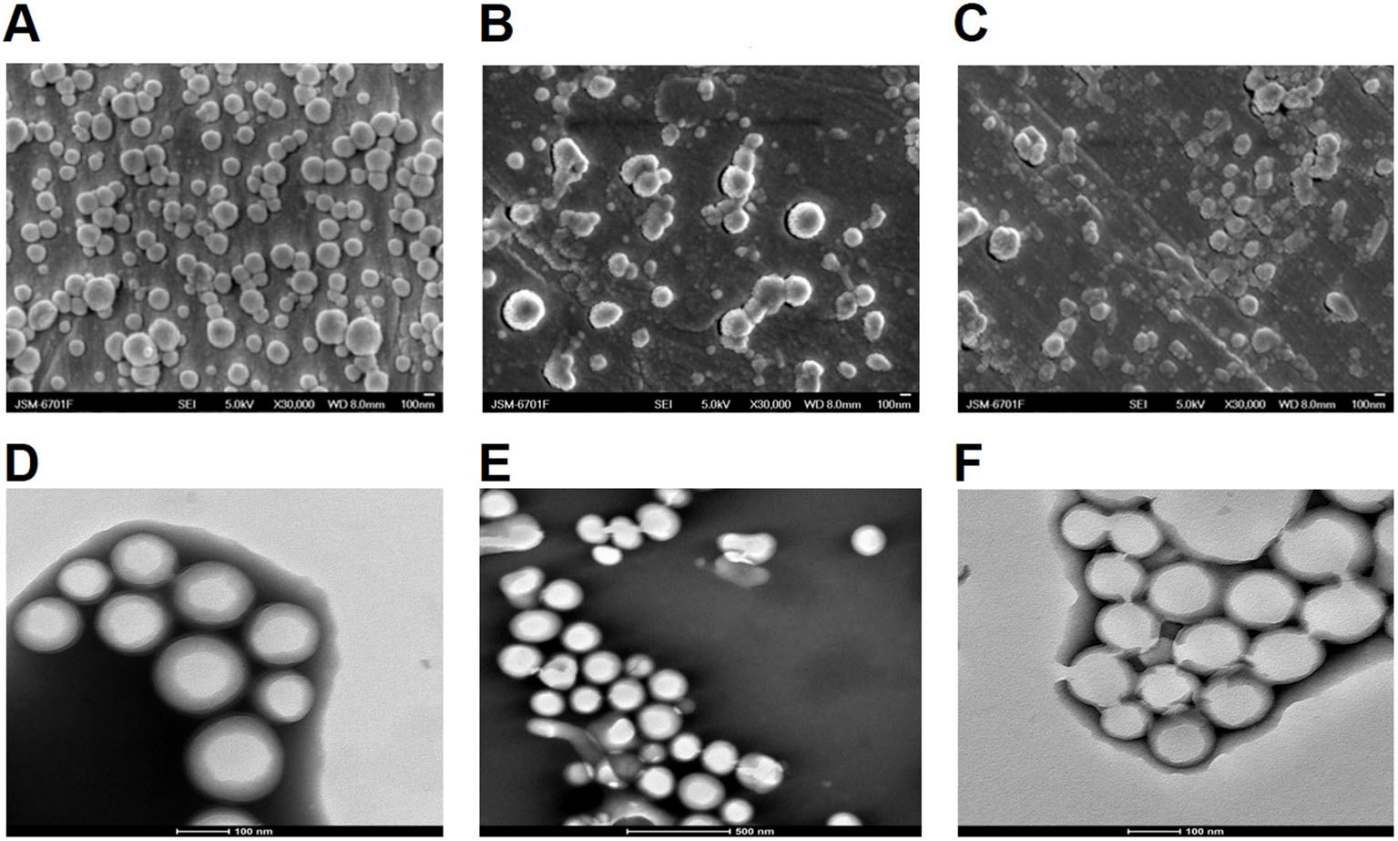
SEM and TEM analysis of PLGA-PEG NPs. The morphology of the NPs was observed using a JSM-6701F model scanning electron microscope (SEM) and 200kV biological cryo-transmission electron microscope (TEM). SEM image of **(A)** OVA/PPS; **(B)** OVA/MPS; **(C)** OVA/GPS. TEM image of **(D)** OVA/PPS; **(E)** OVA/MPS; **(F)** OVA/MPS.

OVA and SR717 were encapsulated in PP, MP, or GP using a double emulsion solvent evaporation method to form NPs. These NPs were characterized by particle size measurements and zeta potential measurements using dynamic light scattering (DLS) at 25°C. The average diameter of these NPs ranged from 200 to 300 nm, and their zeta potentials were all negative. SEM and TEM images revealed that these NPs were spherical and exhibit a relatively uniform size (**Figure 2**). The EE and LP of OVA were calculated by measuring the protein content in the supernatant using a BCA protein assay kit. Similarly, the EE and LP of SR717 were assessed using a LC-MS after dissolving the NPs in DMSO (**Table 1**). These PLGA-PEG NPs showed a relatively higher EE and LP. The EE of OVA reached approximately 60%, with an LP of about 1%. For SR717, the EE was around 60%, with an LP of about 2%.

**Table 1:** Physicochemical characterization of NPs.

Formulations	Size (nm) ± S.D.	PdI ± S.D.	Zeta(mV) ± S.D.	OVA		SR717	
				EE (%)	LP (%)	EE (%)	LP (%)
OVA/PPS	276.86 ± 4.59	0.149 ± 0.03	-13.24 ± 0.31	72	1.4	57.9	2.3
OVA/MPS	211.94 ± 14.9	0.133 ± 0.03	-19.71 ± 0.71	77	1.5	63.6	2.5
OVA/GPS	238.53 ± 12.5	0.148 ± 0.03	-25.20 ± 0.29	60	1.2	62.7	2.5
OVA/PP	337.9 ± 2.86	0.108 ± 0.02	-5.91 ± 0.09	66	1.3	–	–
OVA/MP	285.53 ± 1.79	0.252 ± 0.01	-13.99 ± 0.37	61	1.2	–	–
OVA/GP	270.92 ± 2.81	0.258 ± 0.01	-7.46 ± 0.94	63	1.3	–	–

PdI, polydispersity index; Zeta, zeta potential; EE, encapsulation efficiency; LP, loading percentage; OVA/PPS, OVA/PLGA-PEG-SR717; OVA/MPS, OVA/Man-PLGA-PEG-SR717; OVA/GPS, OVA/Tri-GalNAc-PLGA-PEG-SR717; OVA/PP, OVA/PLGA-PEG; OVA/MP, OVA/Man-PLGA-PEG; OVA/GP, OVA/Tri-GalNAc-PLGA-PEG.

### 3.3 OVA/GP are targeted to lymph nodes *in vivo*

**Figure 3:**
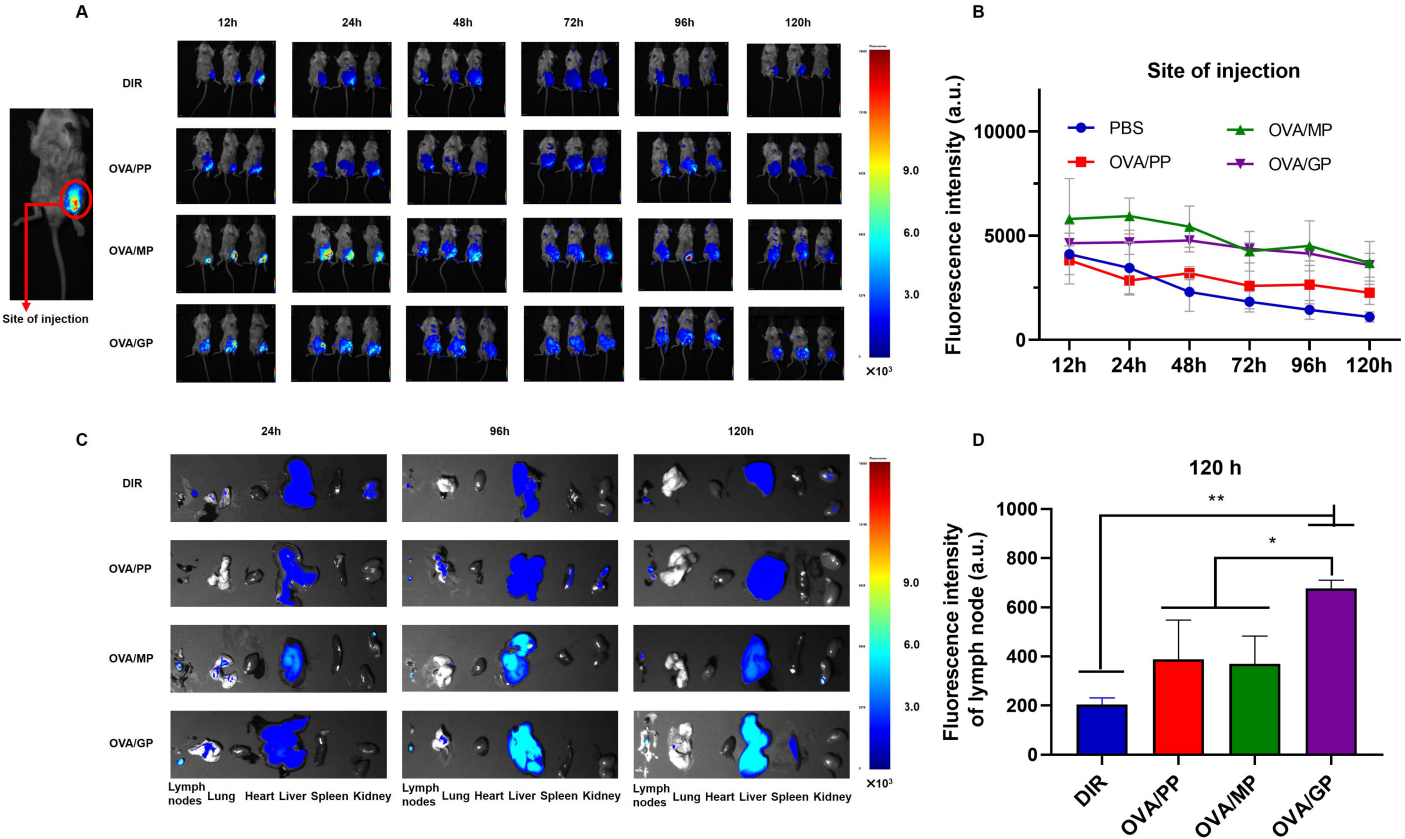
OVA/GP effectively target lymph nodes in mice. **(A)** The images of DIR-NPs in the injection site after injection for 12, 24, 48, 72, 96 and 120h. **(B)** The fluorescent intensity of DIR on the injection site at different time points after injection. **(C)** The images of DIR-NPs in the isolated major organs after injection for 24, 96, and 120 h. **(D)** The fluorescent intensity of DIR in the inguinal lymph nodes at 120 h post-immunization. All images were overlays of bright photographs with fluorescence intensity measurement indicated on the color scale. The results are shown as means ± SD (*n*=3-6), *p < 0.5, **p < 0.01.

To track the delivery of the NPs *in vivo*, the mice were injected subcutaneously in the groin with free DIR in PBS, DIR-labeled OVA/PP(DIR-OVA/PP), DIR-labeled OVA/MP(DIR-OVA/MP), and DIR-labeled OVA/GP(DIR-OVA/GP). The presence of DIR-NPs was visualized at the injection site and in the inguinal lymph node. **Figure 3A** shows representative images of fluorescence signals corresponding to DIR-NPs at the injection site at designated time points. The fluorescence signals at the injection site were quantified and the average fluorescence intensity were plotted over time and presented in **Figure 3B**. The results showed that the fluorescence intensity of free DIR gradually decreased over time. In contrast, the fluorescence intensity of DIR-OVA/PP, DIR-OVA/MP, and DIR-OVA/GP declined slowly. Significant fluorescent signals were observed in the lymph nodes of mice injected with DIR-OVA/MP and DIR-OVA/GP 24h after immunization and persisted until 120h (**Figure 3C**). At 120h post-immunization, the presence of DIR-OVA/GP was also detected in the spleens of mice (**Figure 3C**). A quantitative analysis of the fluorescence intensity in the inguinal lymph nodes 120h post-immunization indicated that DIR-OVA/GP accumulated more in the lymph nodes than free DIR, DIR-OVA/PP, and DIR-OVA/MP (*p*<0.01) (**Figure 3D**). Furthermore, the livers of the mice immunized with NPs exhibited fluorescent signals, with the most intense signals observed in those immunized with the DIR-OVA/GP (**Figure 3C**). 40 h after immunization with DiI-labeled NPs in the inguinal region, the accumulation of OVA/GPS was also detected in the mouse inguinal lymph nodes using flow cytometry (**Supplementary Figure S4**).

### 3.4 OVA/GPS promote the activation of DCs *in vitro*

**Figure 4:**
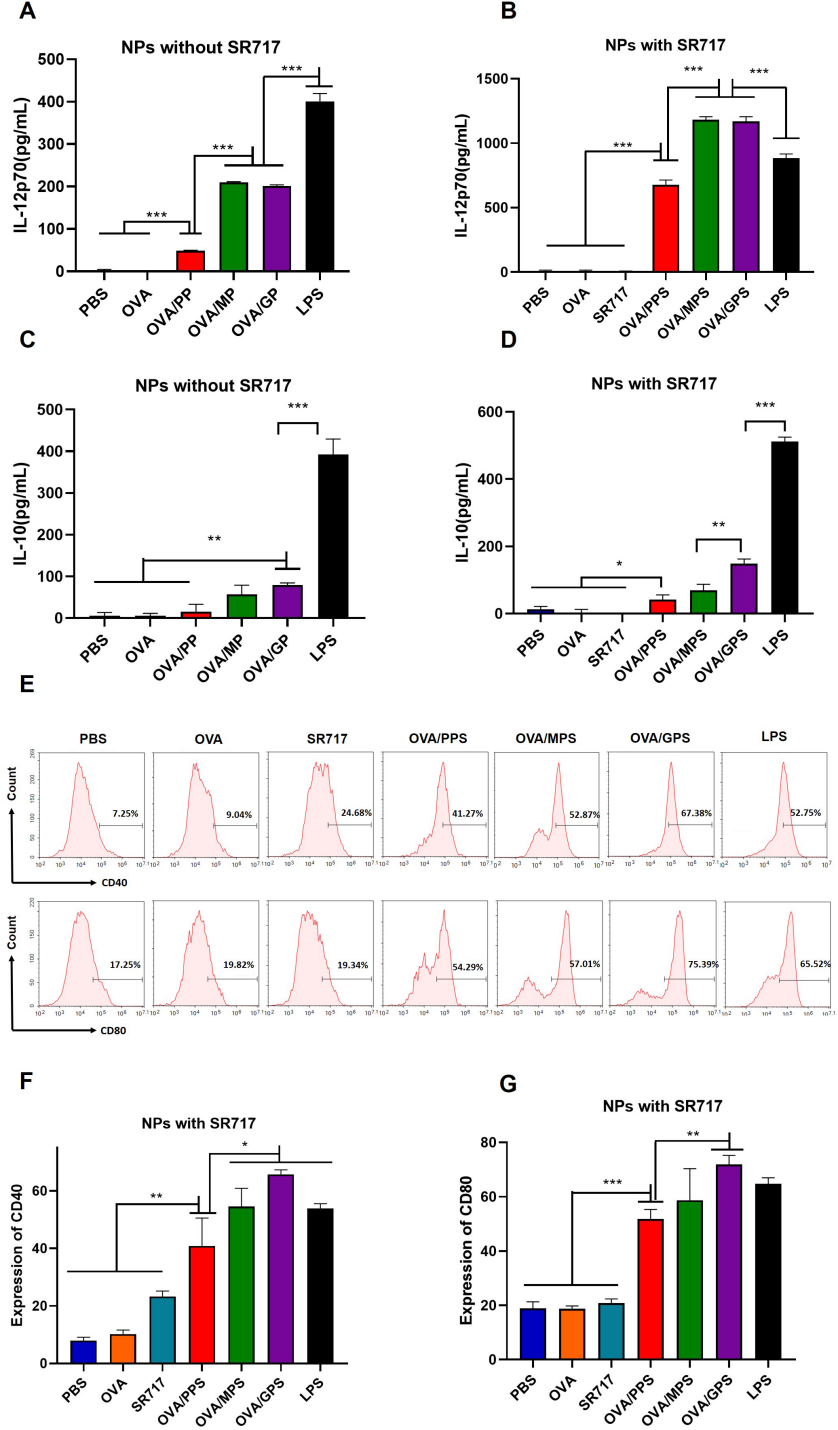
OVA/GPS efficiently activates BMDCs *in vitro*. **(A)** The levels of IL-12p70 produced by BMDCs treated with NPs not encapsulating SR717. **(B)** The levels of IL-12p70 produced by BMDCs treated with NPs encapsulating SR717. **(C)** The levels of IL-10 produced by BMDCs treated with NPs not encapsulating SR717. **(D)** The levels of IL-10 produced by BMDCs treated with NPs encapsulating SR717. **(E)** The expression of CD40 and CD80 on BMDCs treated with NPs encapsulating SR717. **(F, G)** Statistical analysis of the expression of CD40 and CD80 on BMDCs. The results are shown as means ± SD (*n*=3), *p < 0.5, **p < 0.01, ***p < 0.001.

To assess the activation of DCs, the cytokines including IL-12p70 and IL-10 secreted by BMDCs was evaluated after treatment with NPs that were encapsulated with or without SR717 for 24 h. The results indicated that compared to the OVA/PP group, the secretion levels of IL-12p70 in the OVA/MP and OVA/GP groups were increased (*p* < 0.001), but still lower than those in the LPS-stimulated group (*p* < 0.001) (**Figure 4A**). To further promote the maturation of DCs, the agonist SR717 was attempted to encapsulate in NPs. The results indicated that the addition of SR717 significantly increased the IL-12p70 secretion by BMDCs in the OVA/MPS and OVA/GPS groups, exceeding the levels in the LPS group (*p* < 0.001) (**Figure 4B**). Among all group, the LPS induced the highest level of IL-10 secretion (*p* < 0.001) (**Figures 4C, D**). Subsequently, the expression levels of CD40 and CD80 on the surface of BMDCs were assessed. Compared to the PBS, OVA, and SR717, OVA/PPS, OVA/MPS, and OVA/GPS all induced the expression of CD40 and CD80 (*p* < 0.01). Among these, the expression levels of CD40 and CD80 on BMDCs were highest in the OVA/GPS group, with the OVA/PPS and OVA/MPS groups showing the next highest levels (*p* < 0.05) (**Figures 4E–G**). Furthermore, after immunization with OVA/GPS, the expression of CD40 on DCs in mouse inguinal lymph nodes increased compared to the PBS group, as detected by flow cytometry (**Supplementary Figure S5**). The findings indicate that the targeted modification of PLGA-PEG NPs, in conjunction with an agonist, effectively promote the maturation of DCs.

### 3.5 OVA/GPS promote DC’s uptake *in vitro*

**Figure 5:**
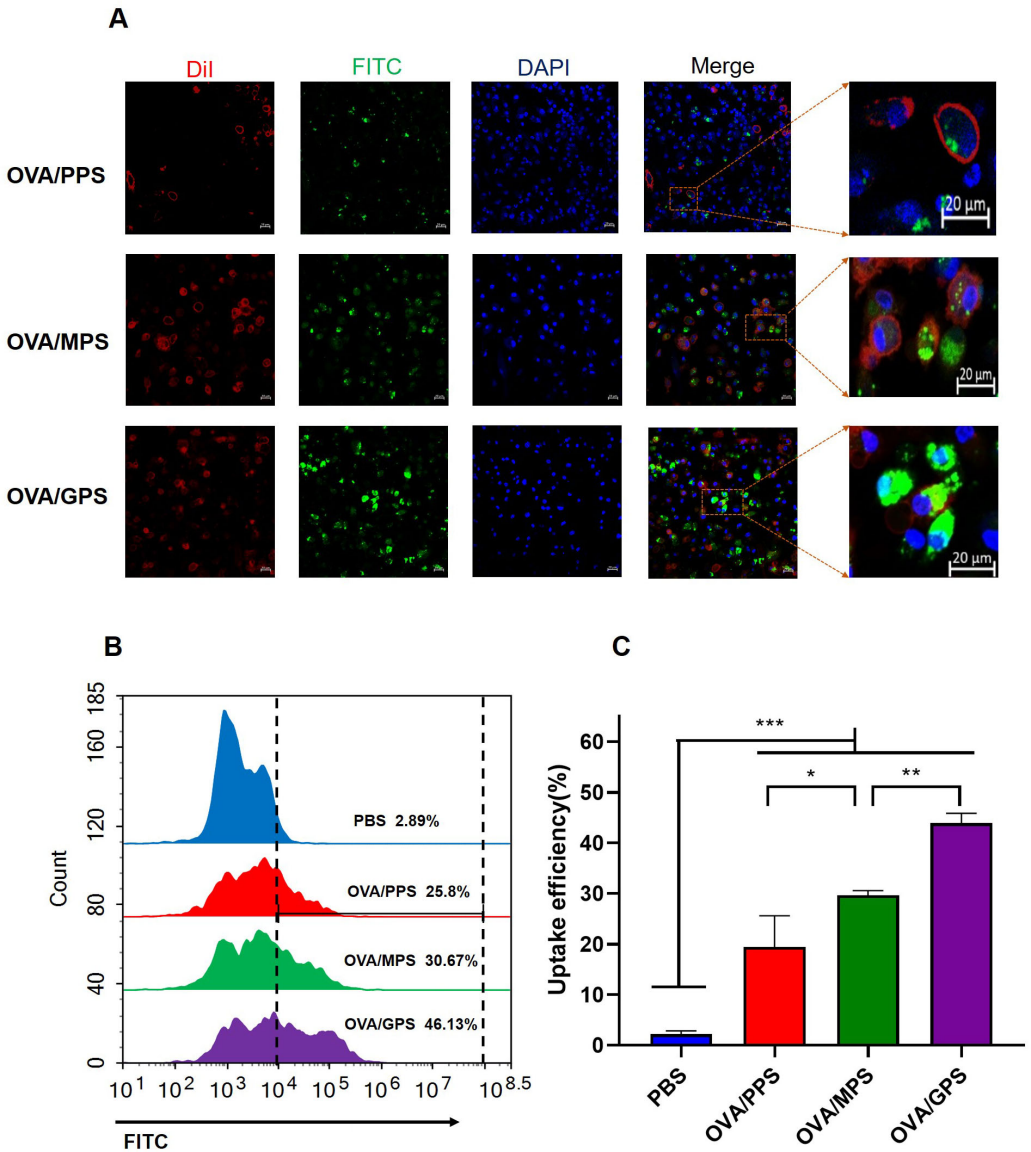
OVA/GPS promote DC’s uptake of antigens *in vitro*. **(A)** The confocal images of BMDCs incubated with FITC-NPs for 3 h at 37°C. The green signal represents FITC-labeled NPs, the red signal indicates DiI-stained cell membrane, and the blue signal depicts DAPI-stained nuclei. **(B)** BMDCs were incubated with FITC-labeled NPs for 3 h, and the histogram of FITC fluorescence intensity of BMDCs was detected by flow cytometry. **(C)** Statistical analysis of the uptake efficiency of NPs by BMDCs through quantification of FITC fluorescence intensity within the BMDCs. The results are shown as means ± SD (*n*=3), *p < 0.5, **p < 0.01, ***p < 0.001.

To assess the antigen delivering effect of NPs to DCs, BMDCs were incubated with FITC-labeled OVA/PPS, OVA/MPS, and OVA/GPS for 3 h. Subsequently, the cell membranes were labeled with DiI and the cell nuclei with DAPI. The internalization of NPs was observed using confocal laser scanning microscopy. At the same time, after incubating with FITC-labeled NPs for 3 h, the degree of BMDCs uptake of NPs was quantified using flow cytometry. As shown in **Figure 5A**, BMDCs exhibit phagocytic activity towards different NPs, with a greater proportion of OVA/GPS being engulfed compared to OVA/PPS and OVA/MPS. Compared to OVA/PPS, the uptake efficiency of BMDCs for OVA/MPS increased by approximately 5% (*p<*0.05); compared to OVA/MPS, the uptake efficiency of BMDCs for OVA/GPS increased by approximately 15% (*p<*0.01) (**Figures 5B, C**). These results suggest that compared to unmodified NPs and Mannose-modified NPs, Tri-GalNAc-modified NPs can significantly increase antigens uptake of BMDCs.

### 3.6 TP/GPS promote the antigen-specific cellular immune responses

Through *in vitro* and *in vivo* experiments, we found that Tri-GalNAc-modified PLGA-PEG NPs encapsulating SR717 effectively targeted and activated DCs. Next, we encapsulated *M. tuberculosis* fusion protein TP in both unmodified and Tri-GalNAc-modified PLGA-PEG NPs and evaluated their ability to induce antigen-specific immune responses.

#### 3.6.1 TP/GPS activate the antigen-specific T cells

**Figure 6:**
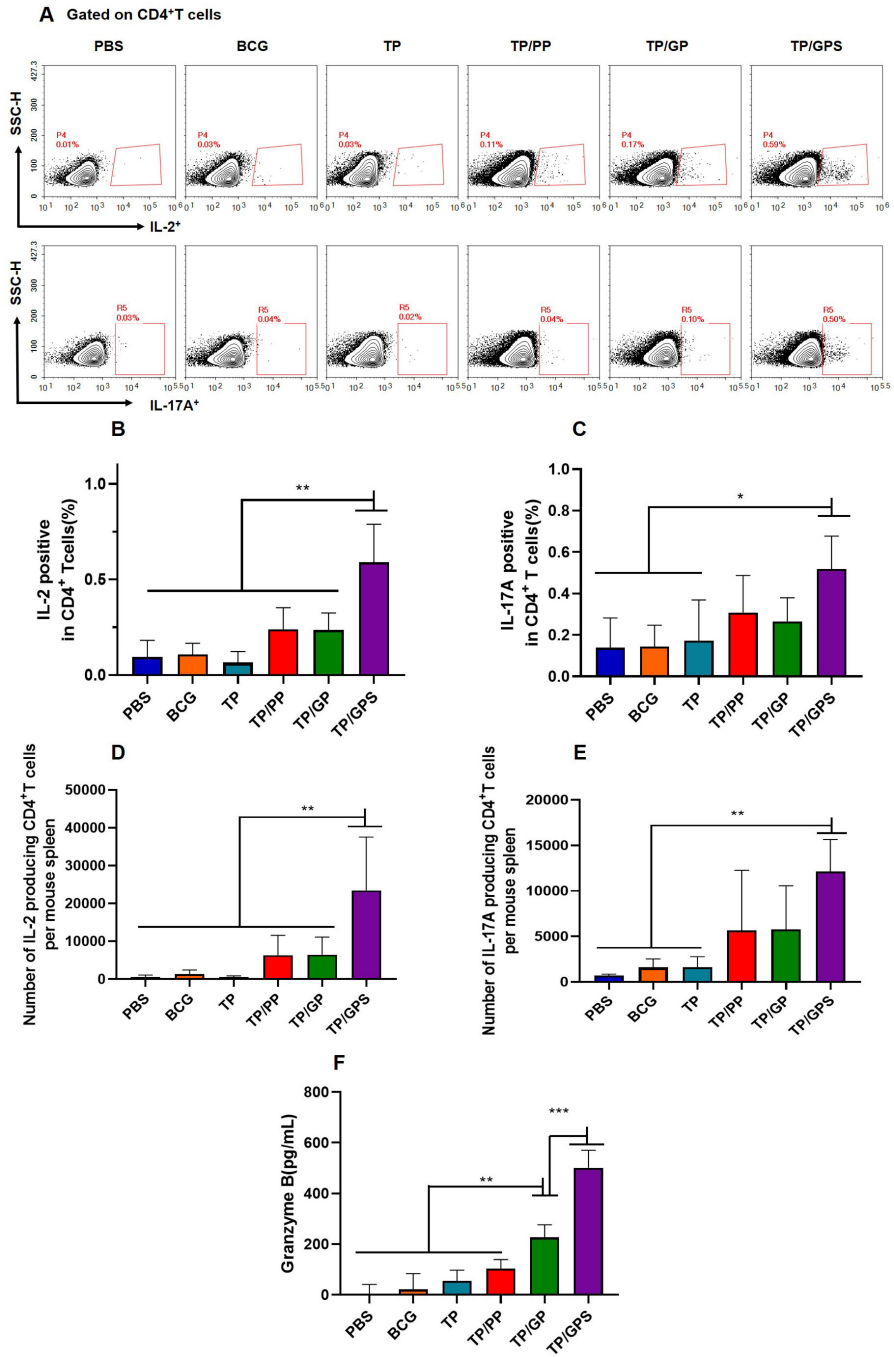
TP/GPS induce the activation of antigen-specific T cells. At 6 weeks after the final immunization, the splenic lymphocytes of mice were isolated and stimulated with individual antigens of TP for 12 h *in vitro*, then the immune responses were analyzed by flow cytometry and ELISA. **(A)** Flow cytometric analysis of IL-2 and IL-17A producing CD4^+^T cells. **(B, C)** Statistical analysis of the proportion of IL-2 and IL-17A producing CD4^+^T cells. **(D-E)** Statistical analysis of the number of IL-2, and IL-17A-producing CD4^+^T cells among total spleen lymphocytes in each mouse. **(F)** The amount of secreted granzyme B from spleen lymphocytes following antigen stimulation. The results are shown as means ± SD (*n*=4-5), *p < 0.5, **p < 0.01, ***p < 0.001.

The cellular immune responses induced by NPs were analyzed at 6 weeks after the final vaccination. The frequencies of IL-2 and IL-17A producing CD4^+^T cells were analyzed by flow cytometry (**Figure 6A**). The results shown that, compared to the PBS, BCG, and TP, the TP/GPS induced higher levels of IL-2 and IL-17A producing CD4^+^T cells (*p*<0.01) (**Figures 6B–E**). Furthermore, the level of Granzyme B secreted by spleen lymphocytes, as measured by ELISA, may indicate the activation of CTLs. As shown in **Figure 6F**, the levels of granzyme B produced from the TP/GPS and TP/GP group were higher than those from PBS, BCG, TP and TP/PP group (*p* < 0.01). The results above demonstrate that PLGA-PEG NPs modified with Tri-GalNAc, encapsulating TP and SR717 effectively induce the generation of antigen-specific CD4^+^ and CD8^+^T cells.

#### 3.6.2 TP/GPS promote the generation of long-term memory CD4^+^ and CD8^+^ T cells

**Figure 7:**
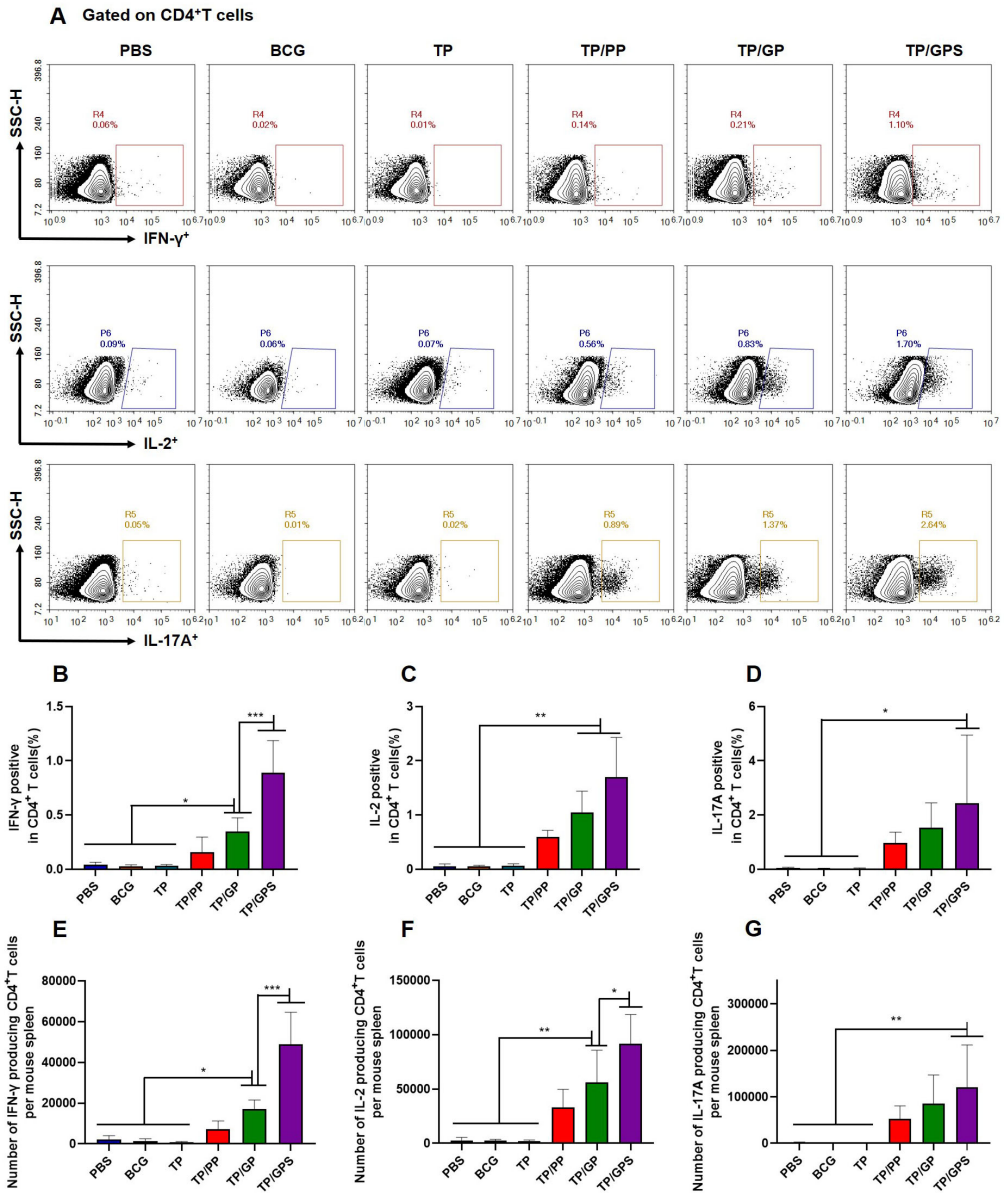
TP/GPS induce the production of antigen-specific memory CD4^+^T cells. At 12 weeks after the final immunization, mice were subcutaneously injected with individual antigens of TP for 3 days. Subsequently, mice were euthanized, and splenic lymphocytes were isolated and stimulated with individual antigens of TP for 12 h *in vitro* before analysis by flow cytometry. **(A)** Flow cytometric analysis of IFN-γ, IL-2, and IL-17A producing CD4^+^T cells. **(B-D)** Statistical analysis of the proportion of IFN-γ, IL-2 and IL-17A producing CD4^+^T cells. **(E-G)** Statistical analysis of the number of IFN-γ, IL-2, and IL-17A producing CD4^+^T cells among total spleen lymphocytes in each mouse. The results are shown as means ± SD (*n*=5), *p < 0.5, **p < 0.01, ***p < 0.001.

To investigate the quantity and quality of memory T cells induced by TP/GPS, the vaccine-induced long-lived memory T cells were evaluated at 12 weeks after last vaccination using the methods as previously mentioned (46). Mice were subcutaneously immunized with individual antigens of TP for *in vivo* stimulation 3 days prior to the immunoassay. After a three-day period, splenic lymphocytes were isolated and stimulated *in vitro* with individual antigens of TP for 12 h. Cytokine secretion by T cells was subsequently assessed using flow cytometry (**Figure 7A**). The results indicated that the TP/GPS group had a higher number of IFN-γ, IL-2, and IL-17A producing CD4^+^ T cells than PBS, BCG, and TP groups. (*p*<0.05) (**Figures 7B–G**). Compared to the TP/GP group, the TP/GPS group showed a significant increase of IFN-γ and IL-2 producing CD4^+^ T cells (**Figures 7B–G**) (*p*<0.05). The results indicate that PLGA-PEG NPs modified with Tri-GalNAc, encapsulating TP and SR717, induce a great number of long-lived CD4^+^ memory T cells.

**Figure 8:**
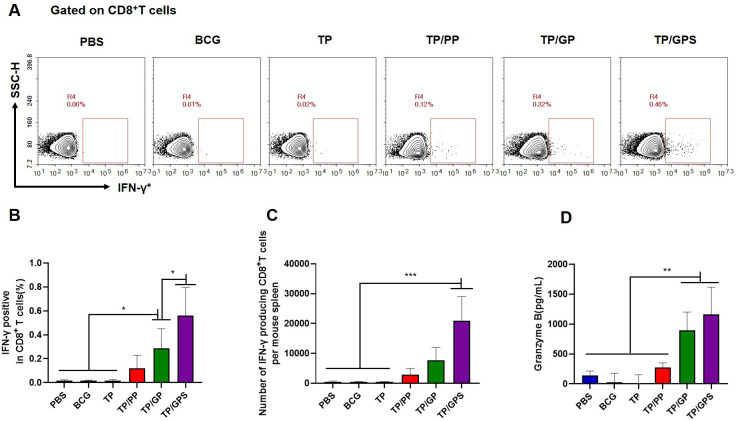
TP/GPS induce the production of antigen-specific memory CD8^+^T cells. **(A)** Flow cytometric analysis of IFN-γ producing CD8^+^T cells. **(B)** Statistical analysis of the proportion of IFN-γ producing CD8^+^T cells. **(C)** Statistical analysis of the number of IFN-γ producing CD8^+^T cells among total spleen lymphocytes in each mouse. **(D)** The amount of secreted granzyme B from spleen lymphocytes following the second antigen stimulation. The results are shown as means ± SD (*n*=5), *p < 0.5, **p < 0.01, ***p < 0.001.

After the second antigenic stimulation, memory CD8^+^T cells differentiate into effector cells that secrete cytotoxic molecules such as granzyme B and cytokines like IFN-γ (48). We assessed the levels of IFN-γ secretion by CD8^+^T cells using flow cytometry (**Figure 8A**) and quantified granzyme B levels in the supernatant with an ELISA kit. The results demonstrate that compared to PBS, BCG, TP, TP/PP, and TP/GP, the TP/GPS significantly elevated numbers of antigen-specific IFN-γ secreting CD8^+^T cells (*p*<0.001) (**Figures 8B, C**). The levels of granzyme B produced from the TP/GPS and TP/GP group were higher than those from PBS, BCG, TP and TP/PP group (*p*<0.01) (**Figure 8D**). The results indicate that PLGA-PEG NPs modified with Tri-GalNAc and SR717 lead to the generation of a substantial number of long-lived CD8^+^ memory T cells.

#### 3.6.3 TP/GPS promote the proliferation of T cells

**Figure 9:**
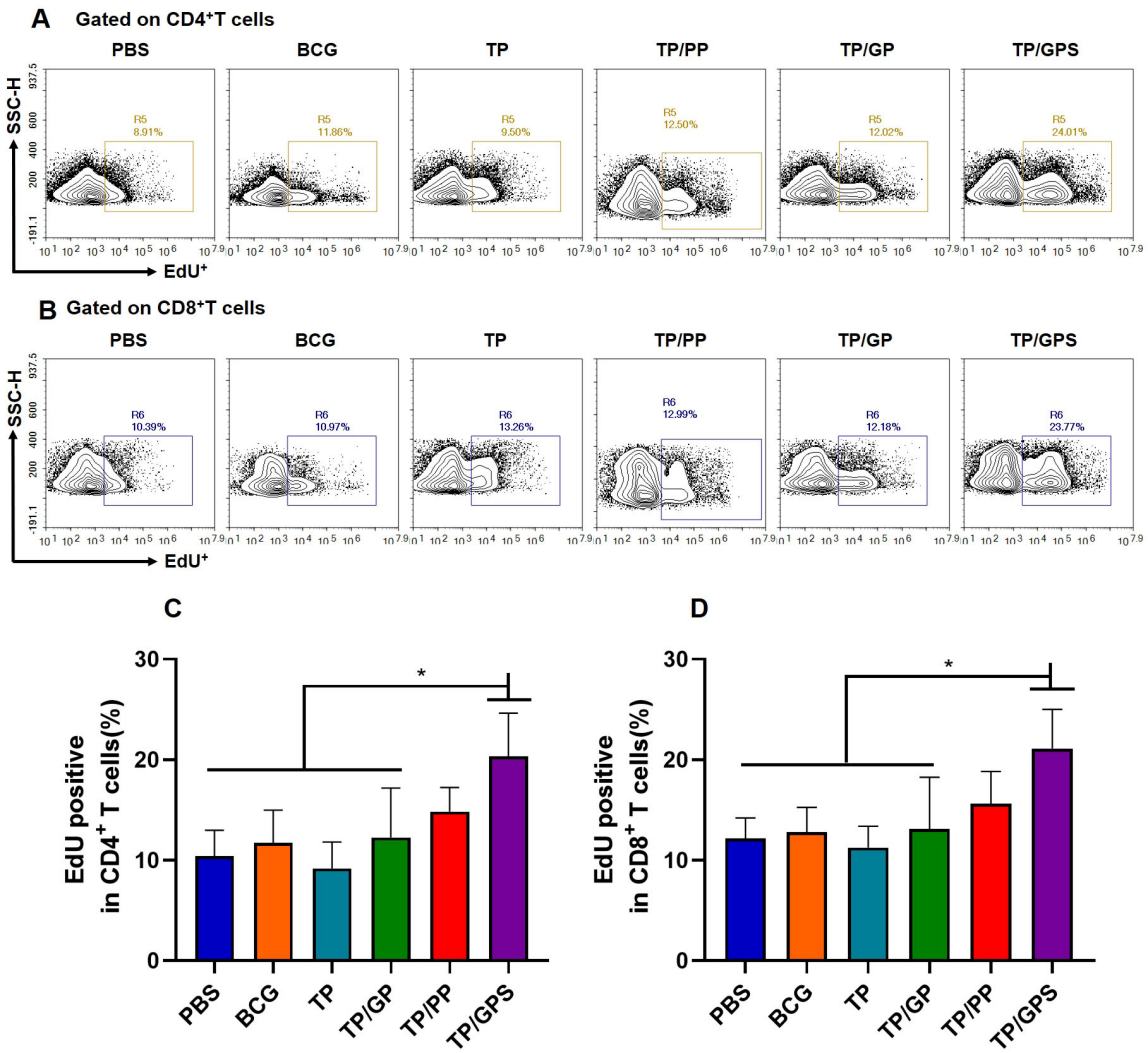
T cell proliferation detected by EdU assay. 12 weeks after the final immunization, mice were subcutaneously injected with individual antigens of TP for 3 days. Following this period, the mice were euthanized, and splenic lymphocytes were isolated and co-cultured with individual antigens of TP and EdU *in vitro* for 3 days prior to flow cytometric analysis. **(A, B)** Representative experiments of flow cytometric analysis of CD4^+^T and CD8^+^T cells proliferation. **(C)** Statistical analysis of CD4^+^T cell proliferation at 12 weeks after the last immunization. **(D)** Statistical analysis of CD8^+^T cell proliferation at 12 weeks after the last immunization. The results are shown as means ± SD (*n*=4), *p < 0.05.

Memory T cells can rapidly proliferate following second antigen stimulation. At 12 weeks after the last immunization, the proliferation capacity of memory T cells were analyzed using the EdU method. Compared to PBS, BCG, TP, and TP/GP groups, the proportion of EdU^+^T cells in the TP/GPS group was significantly increased(*p*<0.05) (**Figure 9**). Taken together, PLGA-PEG NPs modified with Tri-GalNAc, encapsulating TP and SR717 enhance the proliferative capacity of CD4^+^ and CD8^+^ T cells.

### 3.7 TP/GP promote the production of antigen-specific antibodies

**Figure 10:**
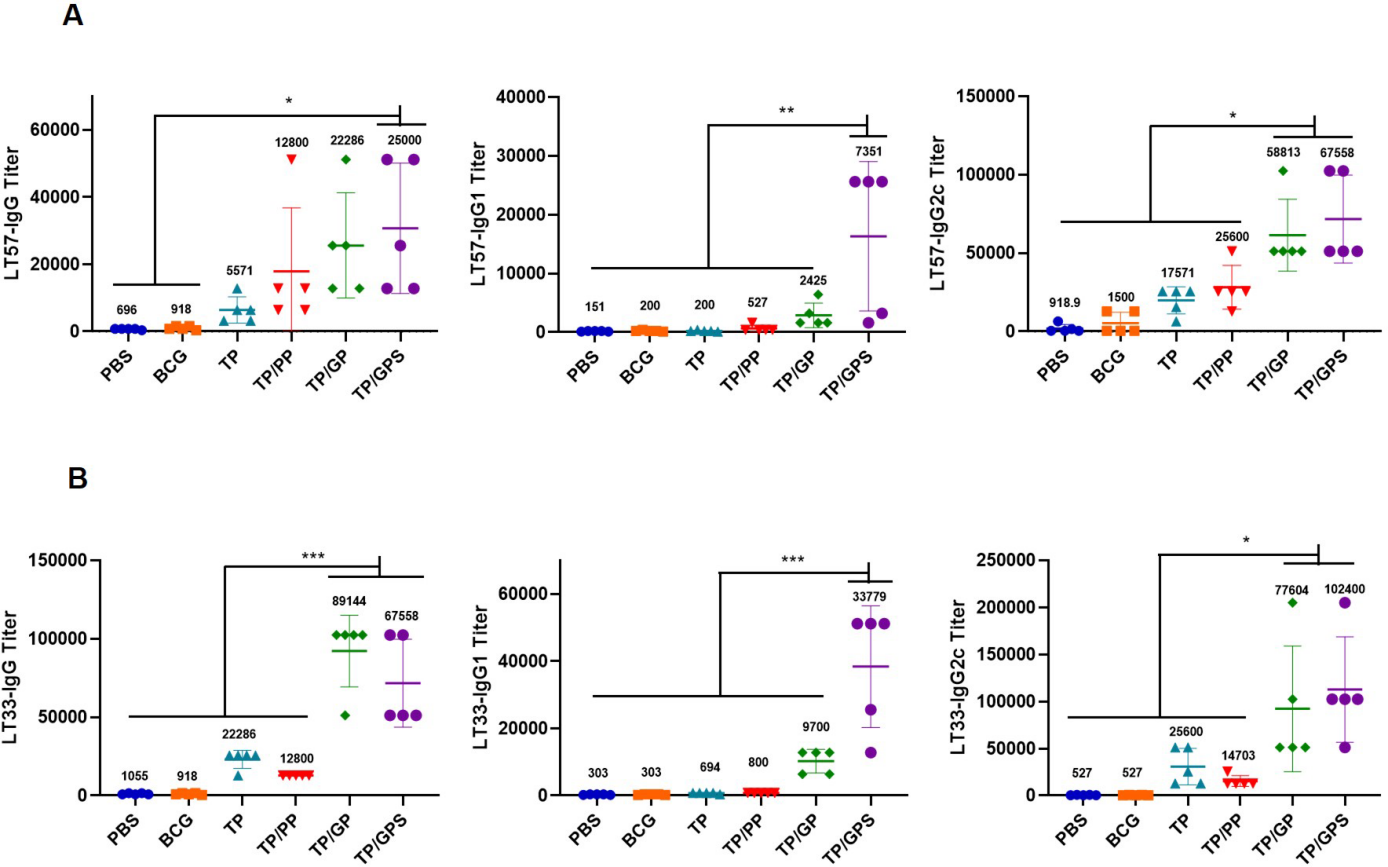
TP/GP induce high levels of antigen-specific antibodies. At 6 weeks after the last immunization, the IgG, IgG1 and IgG2c against LT57 and LT33 in serum were measured by ELISA. **(A)** Statistical analysis of LT57-Specific IgG, IgG1, and IgG2c antibody titers. **(B)** Statistical analysis of LT33-Specific IgG, IgG1, and IgG2c antibody titers. The numbers represent GMT values. (*n*=5). *p < 0.05, **p < 0.01, ***p < 0.001.

At 6 weeks after the last immunization, the LT33 and LT57 specific IgG, IgG1 and IgG2c in serum were measured by ELISA, respectively. The results indicate that, compared to the PBS, BCG, and TP groups, the TP/GP groups exhibit significantly elevated levels of antigen-specific IgG and IgG2c antibodies (*p*<0.05) (**Figure 10**). The results indicate that PLGA-PEG NPs modified with Tri-GalNAc lead to the production of antigen-specific antibodies.

### 3.8 The TP/GPS provides protection against mycobacteria challenge

**Figure 11:**
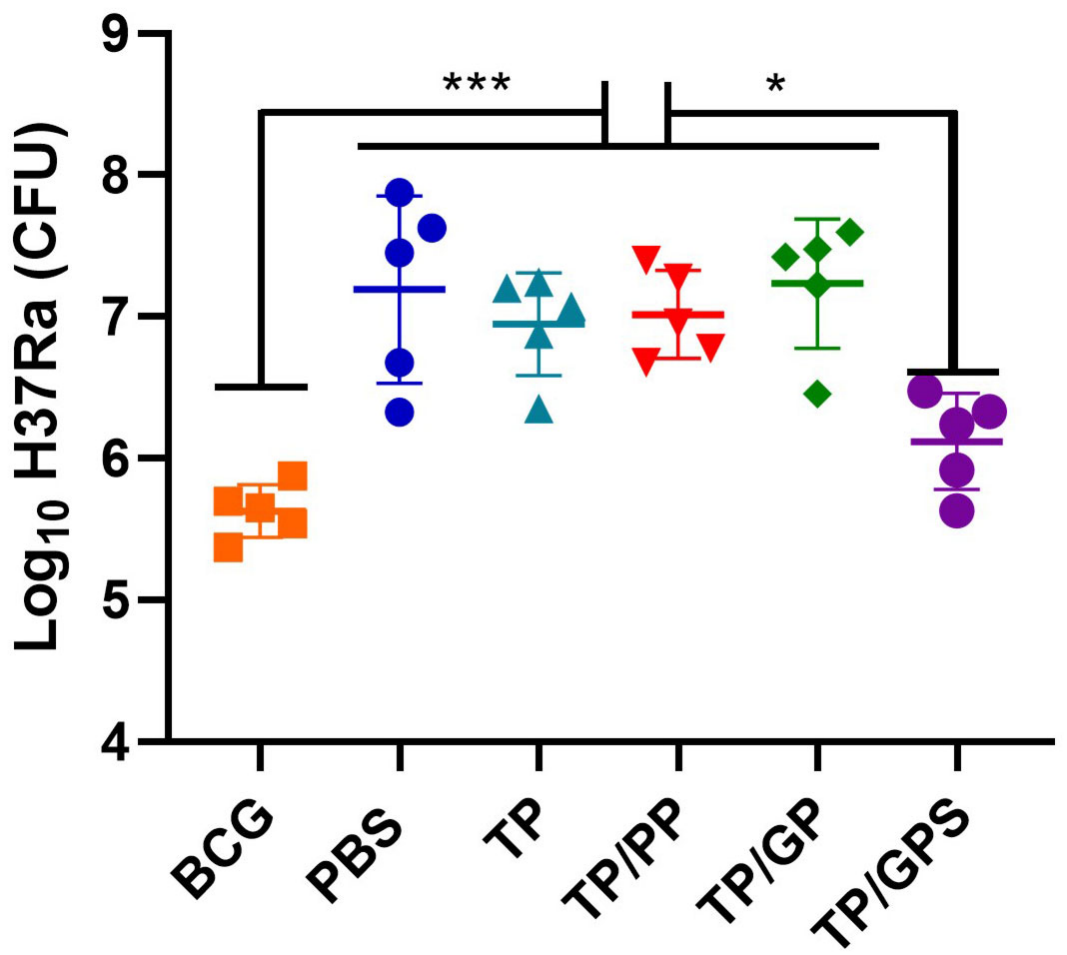
TP/GPS reduces the bacterial load of H37Ra in the Lungs. At the 12 weeks after the last immunization, the C57BL/6 mice were infected intranasally with *M. tuberculosis* H37Ra strain. The protective efficacy was measured by detecting the bacteria load in lung tissues. Mice were euthanized and the bacterial burden (H37Ra) was measured in the lungs. The results are shown as log^10^ CFU ± SD (*n*=5), *p < 0.05, ***p < 0.001.

The high doses of attenuated *M. tuberculosis* H37Ra (5 × 10^6^ CFU per mouse) was used to infect the immunized mice. Considering H37Ra was an attenuated strain and could be cleared in mice around 4 weeks, the bacteria load in lung tissue were determined at 3 weeks after the challenge. The results demonstrated that BCG group and TP/GPS group had a significant reduction in mycobacterial loads in the lungs compared with PBS controls (*p*< 0.05), while other NPs did not show obvious variation (**Figure 11**). It showed that Tri-GalNAc-modified PLGA-PEG NPs co-encapsulated with SR717 promote long-lived memory T cells and improve protective efficacy.

## 4 Discussion

This study developed innovative PLGA-PEG NPs targeted at DCs by modifying them with Tri-GalNAc. The Tri-GalNAc-modified PLGA-PEG NPs effectively delivered antigens to DCs and lymphoid organs. Additionally, the STING agonist SR717 was encapsulated within the NPs to activate DCs and promote a Th1 type cell-mediated immune response. These NPs were further combined with the *M. tuberculosis* fusion protein TP. The Tri-GalNAc-modified PLGA-PEG NPs, combined with SR717 and TP, elicited a robust cellular immune response and offered some protection against mycobacterial infection. In contrast, Tri-GalNAc-modified PLGA-PEG NPs, lacking SR717 induced a strong antigen-specific humoral immune response but did not confer protection against mycobacterial infection.

The modification of carbohydrates on the surface of antigens enhances their uptake and processing by facilitating binding to C-type lectin receptors on DCs (21, 49). In this study, mannose and Tri-GalNAc were separately modified on PLGA-PEG through click chemistry, and double emulsion solvent evaporation method was used to prepare NPs with different sugar modifications. These NPs were found to have low toxicity (**Supplementary Figure S3**). The development of these NPs aims to enhance the targeting of vaccines to DCs, improve the phagocytic efficiency of DCs, and facilitate the delivery of antigens to the draining lymph nodes. Our research found that Tri-GalNAc-modified PLGA-PEG NPs were phagocytosed by DCs 26% more efficiently than unmodified NPs and 14% more efficiently than Mannose-modified NPs. Aggregation of Tri-GalNAc-modified PLGA-PEG NPs were detected in mouse lymph node DCs. Mannose is a monosaccharide that can be recognized by MR on the surface of DCs (50). This recognition mechanism allows mannose-modified NPs to be efficiently engulfed by DCs, thereby enhancing the targeting of the vaccine (51, 52). Existing research has shown that MGL receptor on the surface of immature DCs can specifically recognize and bind to GalNAc-modified antigens, thereby promoting their phagocytosis (22, 53). In our experiment, we modified PLGA-PEG nanoparticles with three GalNAc units. Compared to single GalNAc modification, the decoration of PLGA-PEG NPs with Tri-GalNAc likely leads to better recognition by MGL receptor, resulting in improved uptake by DCs. The MGL receptor is expressed on the surface of monocyte-derived dendritic cells (MoDCs) (54). In diverse inflammatory contexts and types of infections, MoDCs secrete a variety of cytokines and chemokines, leading to the induction of different T cell immune responses (55, 56). The results of this experimental study show that PLGA-PEG NPs modified with Tri-GalNAc, without encapsulating SR717, can elicit a relatively high level of antigen-specific antibody production. When the STING agonist SR717 was added into these NPs, it induced a stronger T cell immune response. We hypothesize that PLGA-PEG NPs modified with Tri-GalNAc may target MoDCs and induce a humoral immune response skewed towards the Th2 phenotype. The addition of SR717 can prompt MoDCs to secrete inflammatory cytokines such as IL-12p70, IFN-I, and IL-23, which then promote the differentiation of CD4^+^T cells into Th1 and Th17 cells (11).

The primary role of an adjuvant is to induce the maturation of DCs to modulate the immune response (57). Targeting antigen to the CLR without a “stimulatory signal” result in the induction of immature DCs, leading to immune tolerance and T-cell unresponsiveness (17, 58). The activation of T cells requires the full maturation of DCs, which is mainly characterized by three key markers: effective antigen presentation, enhanced expression of co-stimulatory molecules, and secretion of inflammatory cytokines (59). Studies have found that DCs which have internalized PLGA NPs without TLR ligands rarely showed an increase in the expression of maturation markers such as CD80, CD86 and CCR7 (60). Our findings indicate that Tri-GalNAc-modified PLGA-PEG NPs, when encapsulating OVA, induce a modest secretion of IL-12p70 by DCs. The non-nucleoside STING agonist SR717 effectively promotes the activation of DCs (35) and elicits a potent innate immune response in the tumor microenvironment (61). When SR717 and OVA were co-encapsulated within these NPs, there was a significant increase in the production of IL-12p70 by DCs. Additionally, these NPs enhance the expression of co-stimulatory molecules such as CD40 and CD80 on the surface of DCs. Cruz et al. also demonstrated that co-encapsulation of the OVA antigen with TLR3 ligand Poly(I:C) and TLR7 ligand R848 within pegylated PLGA NPs, followed by targeted delivery to DCs surface molecules CD40, DEC-205, and CD11c, effectively induced DCs activation (62). Thus, the precise co-delivery of antigen and immunostimulant to the DCs is crucial for more effectively inducing DCs maturation (63).

PLGA-PEG NPs, modified with Tri-GalNAc and encapsulating the *M. tuberculosis* fusion proteins TP and STING agonist SR717, can effectively induce antigen-specific T cell immune responses. It has been reported that after DCs uptake antigens through the MGL receptor, these antigens are delivered to late endosomes for degradation. The resulting peptides are then efficiently presented to CD4^+^T cells *via* MHC-II molecules (64). Recent research also suggests that MUC1 glycopeptides containing Tn antigen can target MHC-I positive compartments, with the potential to activate CD8^+^T cells (65). The binding of SR717 to STING induce the production of type I interferons, which influence the activation of CD4^+^ and CD8^+^T cells (66, 67). Other studies have shown that the co-administration of STING agonists with *M. tuberculosis* fusion proteins in mice can elicit a Th1 and Th17 biased immune response (33, 68). In our study, TP/GPS effectively generated high numbers of IL-2 and IL-17A producing CD4^+^T cells and granzyme B producing CD8^+^T cells. *M. tuberculosis* is an intracellular pathogen, and cellular immune responses play a key role in the prevention of TB (69). Activated CD4^+^T cells can differentiate into various types, including Th1 cells, Th2 cells, Th17 cells, follicular helper T cells (Tfh), and regulatory T cells (Treg) (70). Th1 cells activate macrophages by secreting factors such as IFN-γ and IL-2, enhancing their ability to combat *M. tuberculosis* and promoting the formation of granulomas to control the infection (71). Th2 cells participate in humoral immunity, helping B cells produce antibodies (72). Th17 cells play a role in early anti-tuberculosis infection by secreting factors such as IL-17 (73). Tfh can assist B cells in producing high-affinity antibodies and promote the formation of memory B cells. CD8^+^T cells, also known as CTLs (74), can kill infected cells by secreting cytotoxic molecules like granzymes, and activate macrophages by secreting IFN-γ, enhancing their phagocytosis and killing of *M. tuberculosis* (75). Previous studies have demonstrated that targeting receptors such as the MGL receptor, CD11c, and CD11b on the surface of DCs, in the absence of immunostimulatory, can elicit antibody production (53, 76). Our research indicated that, compared to unmodified NPs, Tri-GalNAc-modified NPs induced higher levels of antigen-specific IgG and IgG2c in mice. However, these NPs failed to induce immune protection against H37Ra strain infection. Therefore, the contribution of antibodies in immune protection against *M. tuberculosis* infection still requires further investigation.

Since TB is a chronic infectious disease, an ideal TB vaccine should provide long-lasting immune protection (5, 77). The persistence of memory T cells is a key factor in achieving durable anti-TB immune protection in the body (2, 5). After vaccination, memory cells are formed in the body. When infection, these memory cells rapidly respond, producing effector cytokines and other antimicrobial molecules to eliminate pathogens (78). In our study, the immune responses induced by memory T cells were evaluated 12 weeks after the final immunization. The results shown that, compared to unmodified NPs (TP/PP) and Tri-GalNAc-modified NPs (TP/GP), TP/GPS induced a high numbers of memory CD4^+^ and CD8^+^T cells, and offers a certain level of protection against H37Ra strain infection. Targeting dendritic cells with NPs efficiently minimizes local inflammation and promotes migration to secondary lymphoid organs (79, 80). Within these organs, the NPs release antigens and agonist, favoring the formation of immune memory (24). In another approach, OVA is chemically conjugated to an antibody specific for the CLR DEC-205, and co-immunization with Poly(I:C), CpG, and c-di-AMP in mice, which effectively induce the generation of memory CD8^+^T cells (81). Zhang and colleagues developed mannosylated lipid-polymer hybrid NPs that integrates a TLR7/8 agonist, imiquimod (IMQ), and a TLR4 agonist, monophosphoryl Lipid A (MPLA). Upon adsorption the OVA antigen onto its surface, this NPs formulation was capable of inducing T cell immune memory (82).

The Tri-GalNAc-modified PLGA-PEG NPs encapsulating SR717 and antigens offers several advantages. Firstly, PLGA NPs have excellent biocompatibility and can slowly release antigens (83). The PEG modification further enhances the hydrophilicity and circulation time of the NPs *in vivo* (84), allowing for better targeting of DCs and reducing initial local inflammatory responses. Secondly, the Tri-GalNAc modification enables the PLGA-PEG NPs to specifically bind to the MGL receptor on DCs, enhancing the uptake and phagocytosis of the NPs by DCs (85). Lastly, the encapsulated antigens and SR717 are effectively protected from degradation *in vivo* (86). Once they reach the DCs, the NPs slowly release the antigen and SR717, inducing the activation of the DCs and initiating an adaptive immune response. These NPs concealing the antigen and SR717 to evade the body’s initial defense mechanisms, ensuring immune activation upon reaching target cells.

PLGA-PEG NPs modified with Tri-GalNAc and encapsulating SR717 and antigens can stimulate a sustained antigen-specific cellular immune response, making them suitable for developing vaccine adjuvants targeting intracellular pathogens such as Brucella, Salmonella, and Legionella pneumonia. In contrast, Tri-GalNAc-modified NPs without SR717 efficiently induce the production of antigen-specific antibodies, which makes them ideal for developing adjuvants for vaccines against disease like influenza, pneumonia, COVID-19, where humoral immune responses are crucial for combating infections. Furthermore, we have found that these Tri-GalNAc modified PLGA-PEG NPs can target the liver, offering a promising new avenue for drug delivery to treat liver-related diseases. Nevertheless, this characteristic may, to some extent, affect the efficacy of vaccines.

In summary, Tri-GalNAc-modified PLGA-PEG NPs function as an efficient antigen delivery system to DCs, with the synergistic effect of SR717 further enhancing DCs activation. When combined with the *M. tuberculosis* fusion protein TP, these NPs induce a robust cellular immune memory response. This suggests that Tri-GalNAc-modified PLGA-PEG NPs could serve as an effective adjuvant for subunit vaccines aimed at inducing long-term T cell-mediated immunity against intracellular pathogens.

## Data Availability

The original contributions presented in the study are included in the article/**Supplementary Material**. Further inquiries can be directed to the corresponding authors.

## References

[1] Pollard AJ Bijker EM. A guide to vaccinology: from basic principles to new developments. Nat Rev Immunol. (2021) 21:83–100. doi: 10.1038/s41577-020-00479-7 33353987 PMC7754704

[2] Zhu B Dockrell HM Ottenhoff THM Evans TG Zhang Y. Tuberculosis vaccines: Opportunities and challenges. Respirol (Carlton Vic). (2018) 23:359–68. doi: 10.1111/resp.2018.23.issue-4 29341430

[3] Dorman SE Holland SM. Interferon-gamma and interleukin-12 pathway defects and human disease. Cytokine Growth Factor Rev. (2000) 11:321–33. doi: 10.1016/S1359-6101(00)00010-1 10959079

[4] Lyadova IV Panteleev AV. Th1 and th17 cells in tuberculosis: protection, pathology, and biomarkers. Mediators Inflamm. (2015) 2015:854507. doi: 10.1155/2015/854507 26640327 PMC4657112

[5] Li F Dang WR Du YJ Xu XA He P Zhou YH. Tuberculosis vaccines and T cell immune memory. Vaccines. (2024) 12(5):483–503. doi: 10.3390/vaccines12050483 PMC11125691 38793734

[6] Gattinoni L Lugli E Ji Y Pos Z Paulos CM Quigley MF. A human memory T cell subset with stem cell-like properties. Nat Med. (2011) 17:1290–7. doi: 10.1038/nm.2446 PMC3192229 21926977

[7] Obar JJ Lefrançois L. Memory CD8+ T cell differentiation. Ann New York Acad Sci. (2010) 1183:251–66. doi: 10.1111/j.1749-6632.2009.05126.x PMC2836783 20146720

[8] Zhao T Cai Y Jiang Y He X Wei Y Yu Y. Vaccine adjuvants: mechanisms and platforms. Signal Transduct Target Ther. (2023) 8:283. doi: 10.1038/s41392-023-01557-7 37468460 PMC10356842

[9] O’Hagan DT De Gregorio E. The path to a successful vaccine adjuvant–’the long and winding road’. Drug Discovery Today. (2009) 14:541–51. doi: 10.1016/j.drudis.2009.02.009 19508916

[10] Qian C Cao X. Dendritic cells in the regulation of immunity and inflammation. Semin Immunol. (2018) 35:3–11. doi: 10.1016/j.smim.2017.12.002 29242034

[11] Anderson DA 3rd Dutertre CA Ginhoux F Murphy KM. Genetic models of human and mouse dendritic cell development and function. Nat Rev Immunol. (2021) 21:101–15. doi: 10.1038/s41577-020-00413-x PMC10955724 32908299

[12] Coffman RL Sher A Seder RA. Vaccine adjuvants: putting innate immunity to work. Immunity. (2010) 33:492–503. doi: 10.1016/j.immuni.2010.10.002 21029960 PMC3420356

[13] Yin X Chen S Eisenbarth SC. Dendritic cell regulation of T helper cells. Annu Rev Immunol. (2021) 39:759–90. doi: 10.1146/annurev-immunol-101819-025146 33710920

[14] Tang X Zhang J Sui D Yang Q Wang T Xu Z. Simultaneous dendritic cells targeting and effective endosomal escape enhance sialic acid-modified mRNA vaccine efficacy and reduce side effects. J Controlled Release: Off J Controlled Release Society. (2023) 364:529–45. doi: 10.1016/j.jconrel.2023.11.008 37949317

[15] Geijtenbeek TB van Vliet SJ Engering A t Hart BA van Kooyk Y. Self- and nonself-recognition by C-type lectins on dendritic cells. Annu Rev Immunol. (2004) 22:33–54. doi: 10.1146/annurev.immunol.22.012703.104558 15032573

[16] Figdor CG van Kooyk Y Adema GJ. C-type lectin receptors on dendritic cells and Langerhans cells. Nat Rev Immunol. (2002) 2:77–84. doi: 10.1038/nri723 11910898

[17] Geijtenbeek TB Gringhuis SI. Signalling through C-type lectin receptors: shaping immune responses. Nat Rev Immunol. (2009) 9:465–79. doi: 10.1038/nri2569 PMC7097056 19521399

[18] Espuelas S Thumann C Heurtault B Schuber F Frisch B. Influence of ligand valency on the targeting of immature human dendritic cells by mannosylated liposomes. Bioconjugate Chem. (2008) 19:2385–93. doi: 10.1021/bc8002524 19053315

[19] Foged C Arigita C Sundblad A Jiskoot W Storm G Frokjaer S. Interaction of dendritic cells with antigen-containing liposomes: effect of bilayer composition. Vaccine. (2004) 22:1903–13. doi: 10.1016/j.vaccine.2003.11.008 15121302

[20] Tsuiji M Fujimori M Ohashi Y Higashi N Onami TM Hedrick SM. Molecular cloning and characterization of a novel mouse macrophage C-type lectin, mMGL2, which has a distinct carbohydrate specificity from mMGL1. J Biol Chem. (2002) 277:28892–901. doi: 10.1074/jbc.M203774200 12016228

[21] van Dinther D Stolk DA van de Ven R van Kooyk Y de Gruijl TD den Haan JMM. Targeting C-type lectin receptors: a high-carbohydrate diet for dendritic cells to improve cancer vaccines. J Leukoc Biol. (2017) 102:1017–34. doi: 10.1189/jlb.5MR0217-059RR PMC5597514 28729358

[22] Gabba A Attariya R Behren S Pett C van der Horst JC Yurugi H. MUC1 glycopeptide vaccine modified with a galNAc glycocluster targets the macrophage galactose C-type lectin on dendritic cells to elicit an improved humoral response. J Am Chem Society. (2023) 145:13027–37. doi: 10.1021/jacs.2c12843 PMC10288512 37279388

[23] Apostolopoulos V Thalhammer T Tzakos AG Stojanovska L. Targeting antigens to dendritic cell receptors for vaccine development. J Drug Delivery. (2013) 2013:869718. doi: 10.1155/2013/869718 PMC3817681 24228179

[24] Chou PY Lin SY Wu YN Shen CY Sheu MT Ho HO. Glycosylation of OVA antigen-loaded PLGA nanoparticles enhances DC-targeting for cancer vaccination. J Controlled Release: Off J Controlled Release Society. (2022) 351:970–88. doi: 10.1016/j.jconrel.2022.10.002 36220488

[25] Pulendran B SA P O’Hagan DT. Emerging concepts in the science of vaccine adjuvants. Nat Rev Drug Discovery. (2021) 20:454–75. doi: 10.1038/s41573-021-00163-y PMC8023785 33824489

[26] Kumar S Sunagar R Gosselin E. Bacterial protein toll-like-receptor agonists: A novel perspective on vaccine adjuvants. Front Immunol. (2019) 10:1144. doi: 10.3389/fimmu.2019.01144 31191528 PMC6549121

[27] Hopfner KP Hornung V. Molecular mechanisms and cellular functions of cGAS-STING signalling. Nat Rev Mol Cell Biol. (2020) 21:501–21. doi: 10.1038/s41580-020-0244-x 32424334

[28] Hu MM Yang Q Xie XQ Liao CY Lin H Liu TT. Sumoylation promotes the stability of the DNA sensor cGAS and the adaptor STING to regulate the kinetics of response to DNA virus. Immunity. (2016) 45:555–69. doi: 10.1016/j.immuni.2016.08.014 27637147

[29] Diamond MS Kinder M Matsushita H Mashayekhi M Dunn GP Archambault JM. Type I interferon is selectively required by dendritic cells for immune rejection of tumors. J Exp Med. (2011) 208:1989–2003. doi: 10.1084/jem.20101158 21930769 PMC3182061

[30] Sun X Liu T Zhao J Xia H Xie J Guo Y. DNA-PK deficiency potentiates cGAS-mediated antiviral innate immunity. Nat Commun. (2020) 11:6182. doi: 10.1038/s41467-020-19941-0 33273464 PMC7712783

[31] Zheng J Mo J Zhu T Zhuo W Yi Y Hu S. Comprehensive elaboration of the cGAS-STING signaling axis in cancer development and immunotherapy. Mol Cancer. (2020) 19:133. doi: 10.1186/s12943-020-01250-1 32854711 PMC7450153

[32] Wang Y Luo J Alu A Han X Wei Y Wei X. cGAS-STING pathway in cancer biotherapy. Mol Cancer. (2020) 19:136. doi: 10.1186/s12943-020-01247-w 32887628 PMC7472700

[33] Ning H Zhang W Kang J Ding T Liang X Lu Y. Subunit vaccine ESAT-6:c-di-AMP delivered by intranasal route elicits immune responses and protects against mycobacterium tuberculosis infection. Front Cell Infection Microbiol. (2021) 11:647220. doi: 10.3389/fcimb.2021.647220 PMC8019782 33829000

[34] Wang J Li P Yu Y Fu Y Jiang H Lu M. Pulmonary surfactant-biomimetic nanoparticles potentiate heterosubtypic influenza immunity. Sci (New York NY). (2020) 367(6480):eaau0810–38. doi: 10.1126/science.aau0810 PMC7432993 32079747

[35] Chin EN Yu C Vartabedian VF Jia Y Kumar M Gamo AM. Antitumor activity of a systemic STING-activating non-nucleotide cGAMP mimetic. Sci (New York NY). (2020) 369:993–9. doi: 10.1126/science.abb4255 32820126

[36] Tian X Ai J Tian X Wei X. cGAS-STING pathway agonists are promising vaccine adjuvants. Med Res Rev. (2024) 44:1768–99. doi: 10.1002/med.22016 38323921

[37] Gu P Wusiman A Wang S Zhang Y Liu Z Hu Y. Polyethylenimine-coated PLGA nanoparticles-encapsulated Angelica sinensis polysaccharide as an adjuvant to enhance immune responses. Carbohydr Polymers. (2019) 223:115128. doi: 10.1016/j.carbpol.2019.115128 31427012

[38] Rocha CV Gonçalves V da Silva MC Bañobre-López M Gallo J. PLGA-based composites for various biomedical applications. Int J Mol Sci. (2022) 23(4):2034–67. doi: 10.3390/ijms23042034 PMC8876940 35216149

[39] Nair JK Willoughby JL Chan A Charisse K Alam MR Wang Q. Multivalent N-acetylgalactosamine-conjugated siRNA localizes in hepatocytes and elicits robust RNAi-mediated gene silencing. J Am Chem Society. (2014) 136:16958–61. doi: 10.1021/ja505986a 25434769

[40] Wei K Peng X Zou F. Folate-decorated PEG-PLGA nanoparticles with silica shells for capecitabine controlled and targeted delivery. Int J Pharmaceut. (2014) 464:225–33. doi: 10.1016/j.ijpharm.2013.12.047 24463073

[41] Du X Tan D Gong Y Zhang Y Han J Lv W. A new poly(I:C)-decorated PLGA-PEG nanoparticle promotes Mycobacterium tuberculosis fusion protein to induce comprehensive immune responses in mice intranasally. Microbial Pathogenesis. (2022) 162:105335. doi: 10.1016/j.micpath.2021.105335 34861347

[42] Jiang X Hao X Jing L Wu G Kang D Liu X. Recent applications of click chemistry in drug discovery. Expert Opin Drug Discovery. (2019) 14:779–89. doi: 10.1080/17460441.2019.1614910 31094231

[43] Mi Y Liang L Xu K Li Q Wang W Dang W. Severe acute respiratory syndrome coronavirus 2 virus-like particles induce dendritic cell maturation and modulate T cell immunity. Front Cell Infection Microbiol. (2022) 12:986350. doi: 10.3389/fcimb.2022.986350 PMC9682005 36439228

[44] Tan D. Preparation and immunogenicity study of the fusion protein LT33 of Mycobacterium tuberculosis. Lanzhou University. (2021) 9. doi: 10.27204/d.cnki.glzhu.2021.002062

[45] Kaveh DA Whelan AO Hogarth PJ. The duration of antigen-stimulation significantly alters the diversity of multifunctional CD4 T cells measured by intracellular cytokine staining. PloS One. (2012) 7:e38926. doi: 10.1371/journal.pone.0038926 22719990 PMC3373578

[46] Lv W He P Ma Y Tan D Li F Xie T. Optimizing the boosting schedule of subunit vaccines consisting of BCG and “Non-BCG” Antigens to induce long-term immune memory. Front Immunol. (2022) 13:862726. doi: 10.3389/fimmu.2022.862726 35493466 PMC9039131

[47] Niu H Bai C Li F Ma L He J Shi X. Pyrazinamide enhances persistence of T-cell memory induced by tuberculosis subunit vaccine LT70. Tuberculosis (Edinburgh Scotland). (2022) 135:102220. doi: 10.1016/j.tube.2022.102220 35679762

[48] Nolte MA Goedhart M Geginat J. Maintenance of memory CD8 T cells: Divided over division. Eur J Immunol. (2017) 47:1875–9. doi: 10.1002/eji.201747249 29114880

[49] Kim D Wu Y Shim G Oh YK. Lipid nanoparticle-mediated lymphatic delivery of immunostimulatory nucleic acids. Pharmaceutics. (2021) 13(4):490–502. doi: 10.3390/pharmaceutics13040490 PMC8103501 33916667

[50] Pei M Xu R Zhang C Wang X Li C Hu Y. Mannose-functionalized antigen nanoparticles for targeted dendritic cells, accelerated endosomal escape and enhanced MHC-I antigen presentation. Colloids Surfaces B Biointerfaces. (2021) 197:111378. doi: 10.1016/j.colsurfb.2020.111378 33010719

[51] Yang R Xu J Xu L Sun X Chen Q Zhao Y. Cancer cell membrane-coated adjuvant nanoparticles with mannose modification for effective anticancer vaccination. ACS Nano. (2018) 12:5121–9. doi: 10.1021/acsnano.7b09041 29771487

[52] Sun B Zhao X Wu Y Cao P Movahedi F Liu J. Mannose-functionalized biodegradable nanoparticles efficiently deliver DNA vaccine and promote anti-tumor immunity. ACS Appl Mater Interfaces. (2021) 13:14015–27. doi: 10.1021/acsami.1c01401 33751882

[53] Jiang PL Lin HJ Wang HW Tsai WY Lin SF Chien MY. Galactosylated liposome as a dendritic cell-targeted mucosal vaccine for inducing protective anti-tumor immunity. Acta Biomater. (2015) 11:356–67. doi: 10.1016/j.actbio.2014.09.019 25242652

[54] Higashi N Fujioka K Denda-Nagai K Hashimoto S Nagai S Sato T. The macrophage C-type lectin specific for galactose/N-acetylgalactosamine is an endocytic receptor expressed on monocyte-derived immature dendritic cells. J Biol Chem. (2002) 277:20686–93. doi: 10.1074/jbc.M202104200 11919201

[55] Qu C Brinck-Jensen NS Zang M Chen K. Monocyte-derived dendritic cells: targets as potent antigen-presenting cells for the design of vaccines against infectious diseases. Int J Infect Dis. (2014) 19:1–5. doi: 10.1016/j.ijid.2013.09.023 24216295

[56] Segura E Amigorena S. Inflammatory dendritic cells in mice and humans. Trends Immunol. (2013) 34:440–5. doi: 10.1016/j.it.2013.06.001 23831267

[57] Shi S Zhu H Xia X Liang Z Ma X Sun B. Vaccine adjuvants: Understanding the structure and mechanism of adjuvanticity. Vaccine. (2019) 37:3167–78. doi: 10.1016/j.vaccine.2019.04.055 31047671

[58] Macri C Dumont C Johnston AP Mintern JD. Targeting dendritic cells: a promising strategy to improve vaccine effectiveness. Clin Transl Immunol. (2016) 5:e66. doi: 10.1038/cti.2016.6 PMC4815026 27217957

[59] Heras-Murillo I Adán-Barrientos I Galán M Wculek SK Sancho D. Dendritic cells as orchestrators of anticancer immunity and immunotherapy. Nat Rev Clin Oncol. (2024) 21:257–77. doi: 10.1038/s41571-024-00859-1 38326563

[60] Cruz LJ Tacken PJ Pots JM Torensma R Buschow SI Figdor CG. Comparison of antibodies and carbohydrates to target vaccines to human dendritic cells via DC-SIGN. Biomaterials. (2012) 33:4229–39. doi: 10.1016/j.biomaterials.2012.02.036 22410170

[61] Wang B Tang M Yuan Z Li Z Hu B Bai X. Targeted delivery of a STING agonist to brain tumors using bioengineered protein nanoparticles for enhanced immunotherapy. Bioact Mater. (2022) 16:232–48. doi: 10.1016/j.bioactmat.2022.02.026 PMC8965725 35386310

[62] Cruz LJ Rosalia RA Kleinovink JW Rueda F Löwik CW Ossendorp F. Targeting nanoparticles to CD40, DEC-205 or CD11c molecules on dendritic cells for efficient CD8(+) T cell response: a comparative study. J Controlled Release: Off J Controlled Release Society. (2014) 192:209–18. doi: 10.1016/j.jconrel.2014.07.040 25068703

[63] Garg AD Coulie PG Van den Eynde BJ Agostinis P. Integrating next-generation dendritic cell vaccines into the current cancer immunotherapy landscape. Trends Immunol. (2017) 38:577–93. doi: 10.1016/j.it.2017.05.006 28610825

[64] van Vliet SJ Aarnoudse CA Broks-van den Berg VC Boks M Geijtenbeek TB van Kooyk Y. MGL-mediated internalization and antigen presentation by dendritic cells: a role for tyrosine-5. Eur J Immunol. (2007) 37:2075–81. doi: 10.1002/eji.200636838 17616966

[65] Napoletano C Rughetti A Agervig Tarp MP Coleman J Bennett EP Picco G. Tumor-associated Tn-MUC1 glycoform is internalized through the macrophage galactose-type C-type lectin and delivered to the HLA class I and II compartments in dendritic cells. Cancer Res. (2007) 67:8358–67. doi: 10.1158/0008-5472.CAN-07-1035 17804752

[66] Li S Mirlekar B Johnson BM Brickey WJ Wrobel JA Yang N. STING-induced regulatory B cells compromise NK function in cancer immunity. Nature. (2022) 610:373–80. doi: 10.1038/s41586-022-05254-3 PMC9875944 36198789

[67] Zhou Q Dutta D Cao Y Ge Z. Oxidation-responsive polyMOF nanoparticles for combination photodynamic-immunotherapy with enhanced STING activation. ACS Nano. (2023) 17:9374–87. doi: 10.1021/acsnano.3c01333 37141569

[68] Van Dis E Sogi KM Rae CS Sivick KE Surh NH Leong ML. STING-Activating Adjuvants Elicit a Th17 Immune Response and Protect against Mycobacterium tuberculosis Infection. Cell Rep. (2018) 23:1435–47. doi: 10.1016/j.celrep.2018.04.003 PMC6003617 29719256

[69] Gong W Liang Y Wu X. The current status, challenges, and future developments of new tuberculosis vaccines. Hum Vaccines Immunotherapeut. (2018) 14:1697–716. doi: 10.1080/21645515.2018.1458806 PMC6067889 29601253

[70] Josefowicz SZ Lu LF Rudensky AY. Regulatory T cells: mechanisms of differentiation and function. Annu Rev Immunol. (2012) 30:531–64. doi: 10.1146/annurev.immunol.25.022106.141623 PMC6066374 22224781

[71] Flynn JL Chan J. Immune cell interactions in tuberculosis. Cell. (2022) 185:4682–702. doi: 10.1016/j.cell.2022.10.025 PMC12162144 36493751

[72] Abebe F. Synergy between Th1 and Th2 responses during Mycobacterium tuberculosis infection: A review of current understanding. Int Rev Immunol. (2019) 38:172–9. doi: 10.1080/08830185.2019.1632842 31244354

[73] Miossec P Kolls JK. Targeting IL-17 and TH17 cells in chronic inflammation. Nat Rev Drug Discovery. (2012) 11:763–76. doi: 10.1038/nrd3794 23023676

[74] Cheng H Ji Z Wang Y Li S Tang T Wang F. Mycobacterium tuberculosis produces D-serine under hypoxia to limit CD8(+) T cell-dependent immunity in mice. Nat Microbiol. (2024) 9:1856–72. doi: 10.1038/s41564-024-01701-1 PMC11222154 38806671

[75] Kaufmann SHE. Vaccination against tuberculosis: revamping BCG by molecular genetics guided by immunology. Front Immunol. (2020) 11:316. doi: 10.3389/fimmu.2020.00316 32174919 PMC7056705

[76] White AL Tutt AL James S Wilkinson KA Castro FV Dixon SV. Ligation of CD11c during vaccination promotes germinal centre induction and robust humoral responses without adjuvant. Immunology. (2010) 131:141–51. doi: 10.1111/j.1365-2567.2010.03285.x PMC2966766 20465572

[77] Andersen P Kaufmann SH. Novel vaccination strategies against tuberculosis. Cold Spring Harb Perspect Med. (2014) 4(6):a018523–42. doi: 10.1101/cshperspect.a018523 PMC4031959 24890836

[78] Sallusto F Lanzavecchia A Araki K Ahmed R. From vaccines to memory and back. Immunity. (2010) 33:451–63. doi: 10.1016/j.immuni.2010.10.008 PMC3760154 21029957

[79] Kastenmüller W Kastenmüller K Kurts C Seder RA. Dendritic cell-targeted vaccines–hope or hype? Nat Rev Immunol. (2014) 14:705–11. doi: 10.1038/nri3727 25190285

[80] Andersen P Urdahl KB. TB vaccines; promoting rapid and durable protection in the lung. Curr Opin Immunol. (2015) 35:55–62. doi: 10.1016/j.coi.2015.06.001 26113434 PMC4641675

[81] Volckmar J Knop L Stegemann-Koniszewski S Schulze K Ebensen T Guzmán CA. The STING activator c-di-AMP exerts superior adjuvant properties than the formulation poly(I:C)/CpG after subcutaneous vaccination with soluble protein antigen or DEC-205-mediated antigen targeting to dendritic cells. Vaccine. (2019) 37:4963–74. doi: 10.1016/j.vaccine.2019.07.019 31320219

[82] Zhang L Wu S Qin Y Fan F Zhang Z Huang C. Targeted codelivery of an antigen and dual agonists by hybrid nanoparticles for enhanced cancer immunotherapy. Nano Lett. (2019) 19:4237–49. doi: 10.1021/acs.nanolett.9b00030 30868883

[83] Danhier F Ansorena E Silva JM Coco R Le Breton A Préat V. PLGA-based nanoparticles: an overview of biomedical applications. J Controlled Release: Off J Controlled Release Society. (2012) 161:505–22. doi: 10.1016/j.jconrel.2012.01.043 22353619

[84] Cruz LJ Tacken PJ Fokkink R Figdor CG. The influence of PEG chain length and targeting moiety on antibody-mediated delivery of nanoparticle vaccines to human dendritic cells. Biomaterials. (2011) 32:6791–803. doi: 10.1016/j.biomaterials.2011.04.082 21724247

[85] Boscardin SB Hafalla JC Masilamani RF Kamphorst AO Zebroski HA Rai U. Antigen targeting to dendritic cells elicits long-lived T cell help for antibody responses. J Exp Med. (2006) 203:599–606. doi: 10.1084/jem.20051639 16505139 PMC2118236

[86] Verma SK Mahajan P Singh NK Gupta A Aggarwal R Rappuoli R. New-age vaccine adjuvants, their development, and future perspective. Front Immunol. (2023) 14:1043109. doi: 10.3389/fimmu.2023.1043109 36911719 PMC9998920

