# Corrigendum: Enhancing cell-mediated immunity through dendritic cell activation: the role of Tri-GalNAc-modified PLGA-PEG nanoparticles encapsulating SR717

**DOI:** 10.3389/fimmu.2025.1590490

**Published:** 2025-04-30

**Authors:** Yang Gong, Hongbin Jia, Wenrui Dang, Ting Zhou, Pu He, Xiaolei Wang, Bingdong Zhu

**Affiliations:** ^1^ State Key Laboratory for Animal Disease Control and Prevention & Lanzhou Center for Tuberculosis Research, Institute of Pathogen Biology, School of Basic Medical Sciences, Lanzhou University, Lanzhou, China; ^2^ State Key Laboratory of Applied Organic Chemistry, College of Chemistry and Chemical Engineering, Lanzhou University, Lanzhou, China; ^3^ College of Veterinary Medicine, Lanzhou University, Lanzhou, China

**Keywords:** adjuvant, dendritic cells, Tri-GalNAc, SR717, PLGA-PEG nanoparticles, tuberculosis

In the published article, there was an error in **Supplementary Figure S1**. A portion of the content in **Supplementary Figure S1** is missing.

In the published article, there was an error in **Supplementary Figure S2**. A portion of the content in **Supplementary Figure S2** is incorrect.

In the published article, there was an error in **Supplementary Figure S4**. The group names in both the image and the figure legend of **Supplementary Figure S4** are incorrect.

In the published article, there was an error in **Supplementary Figure S5**. The group names in both the image and the figure legend of **Supplementary Figure S5** are incorrect.

The authors apologize for these errors and state that they do not change the scientific conclusions of the article in any way. The original article has been updated.

