# Supplementary material for: Enhancing cell-mediated immunity through dendritic cell activation: the role of Tri-GalNAc-modified PLGA-PEG nanoparticles encapsulating SR717
Source: Front Immunol. 2024 Dec 23;15:1490003. doi: 10.3389/fimmu.2024.1490003 (PMC11701044; doi:10.3389/fimmu.2024.1490003)
Supplement: Supplementary file 1 [file DataSheet1.docx]

**Supporting Information**

**Enhancing Cell-Mediated Immunity through Dendritic Cell Activation: The Role of Tri-GalNAc-Modified PLGA-PEG Nanoparticles Encapsulating SR717**

Yang Gong^1‡^, Hongbin Jia^2‡^, Wenrui Dang^1^, Ting Zhou^1^, Pu He^1^, Xiaolei Wang^2,3*^, Bingdong Zhu^1, 3*^

^1^ State Key Laboratory for Animal Disease Control and Prevention & Lanzhou Center for Tuberculosis Research, Institute of Pathogen Biology, School of Basic Medical Sciences, Lanzhou University, Lanzhou, 730000, China

^2^ State Key Laboratory of Applied Organic Chemistry, College of Chemistry and Chemical Engineering, Lanzhou University, Lanzhou, P. R. China

^3^College of Veterinary Medicine, Lanzhou University, Lanzhou 730000, China.

‡These authors contributed equally to this work

^*^ Corresponding authors:

Bingdong Zhu, Institute of Pathogen Biology, School of Basic Medical Sciences, Lanzhou University. 199 West Donggang Road, Lanzhou 730000, China. E-mail address: [bdzhu@lzu.edu.cn](mailto:bdzhu@lzu.edu.cn).

Xiaolei Wang, State Key Laboratory of Applied Organic Chemistry, College of Chemistry and Chemical Engineering, Lanzhou University, 222 South Tianshui Road, Lanzhou 730000, China. E-mail address: wangxiaolei@lzu.edu.cn

Supplementary Methods

Methods S1: Preparation of DiR-labeled NPs

OVA (1 mg) was dissolved in 1 mL PBS (aqueous phase), while DiR(2mg) and 50 mg PP, MP, or GP were dissolved in 5 mL DCM (organic phase), respectively. Both solutions were sonicated in a 950 W ultrasonic processor for 4 min at 500 W in an ice bath using a 2-mm stepped microhead. The secondary emulsion was further emulsified with the aqueous phase containing 2% (w/v) PVA for 8 min using high pressure homogenizer at 400 bar. The final emulsion was stirred for 4 h to evaporate any DCM. Finally, the NPs were collected by centrifugation at 12,000 × rpm for 20 min and washed two times.

Methods S2: Preparation of FITC-labeled NPs

OVA (1 mg) was dissolved in 1 mL PBS (aqueous phase), while SR717 (2mg), FITC (2mg), and 50 mg PP, MP, or GP were dissolved in 5 mL DCM (organic phase), respectively. Both solutions were sonicated in a 950 W ultrasonic processor for 4 min at 500 W in an ice bath using a 2-mm stepped microhead. The secondary emulsion was further emulsified with the aqueous phase containing 2% (w/v) PVA for 8 min using high pressure homogenizer at 400 bar. The final emulsion was stirred for 4 h to evaporate any DCM. Finally, the NPs were collected by centrifugation at 12,000 × rpm for 20 min and washed two times.

Supplementary Figures


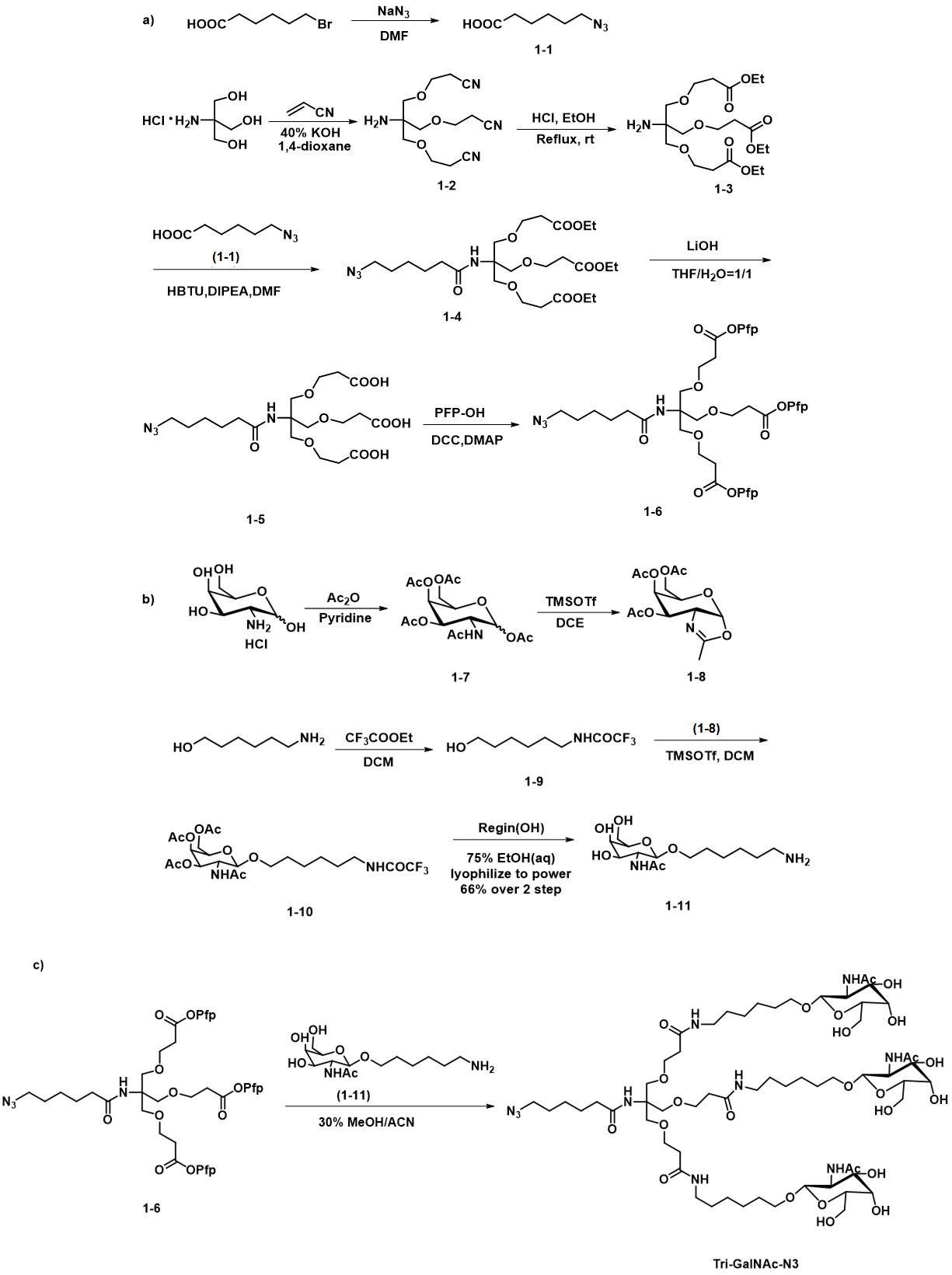


**Figure S1 Synthetic route of the Tri-GalNAc-N3.**


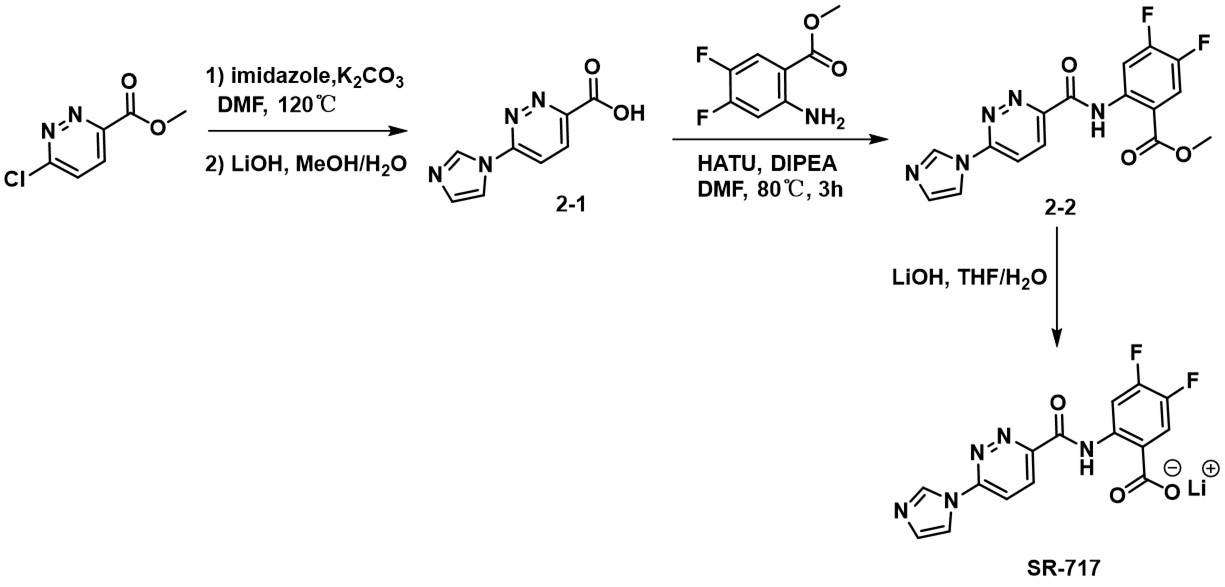


**Figure S2 Synthetic route of the SR717.**


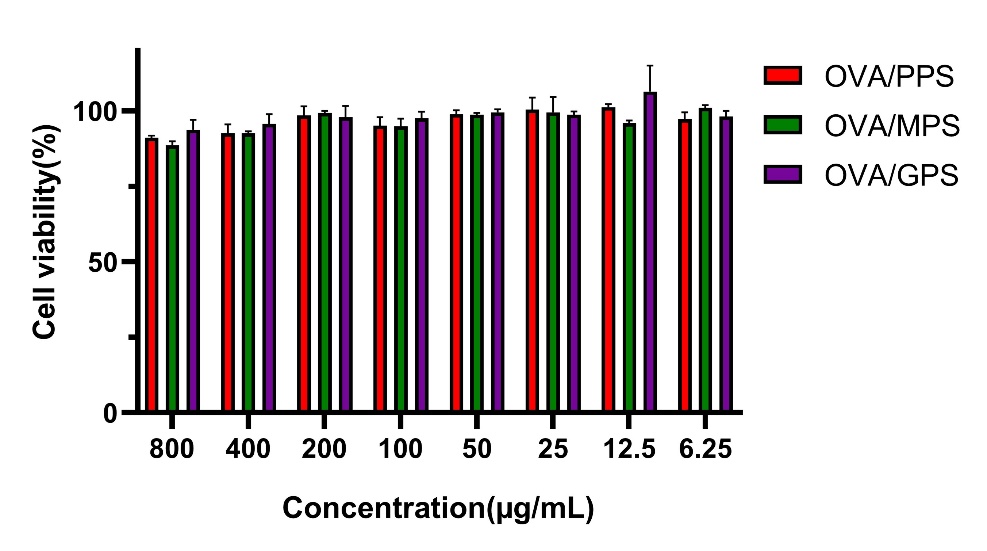


**Figure S3 Effects of NPs on the growth of BMDCs.** The cell viability of BMDCs measured by CCK8 assay after 24 h incubation with NPs. The results are shown as means ± SD (*n*=3).


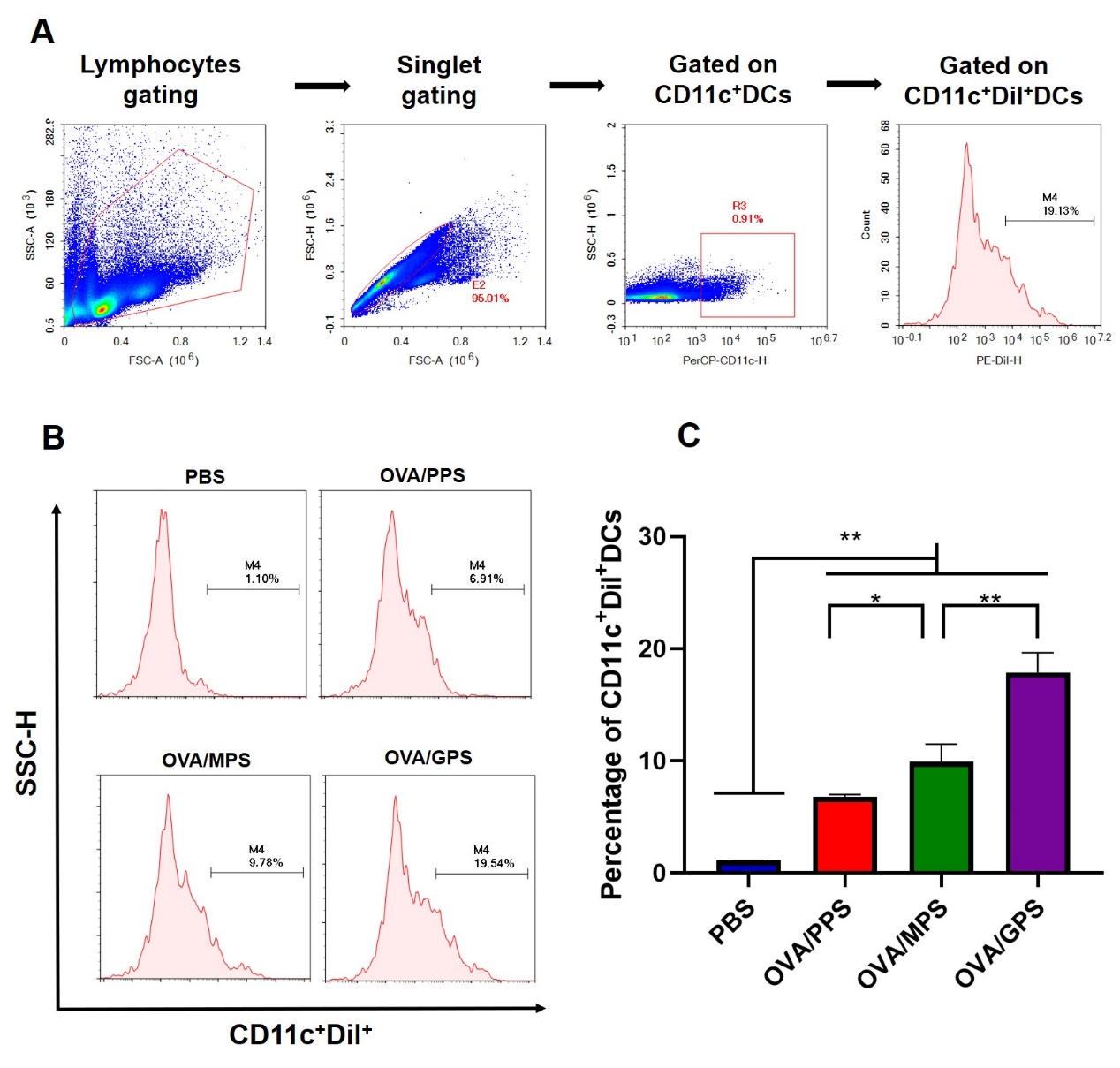


**Figure S4 OVA/GPS target DCs in lymph nodes.** (A) Gating strategy and the representative ﬂow cytometric quantification of DiI positive cells in lymph nodes. (B) Representative flow cytometry dot plot showing DiI positive DCs in lymph node 40 h after subcutaneous injection. (C) Statistics analysis of DiI^+^DCs in lymph node. The results are shown as mean ± SD (*n*=3), **p* < 0.5, ***p* < 0.01.


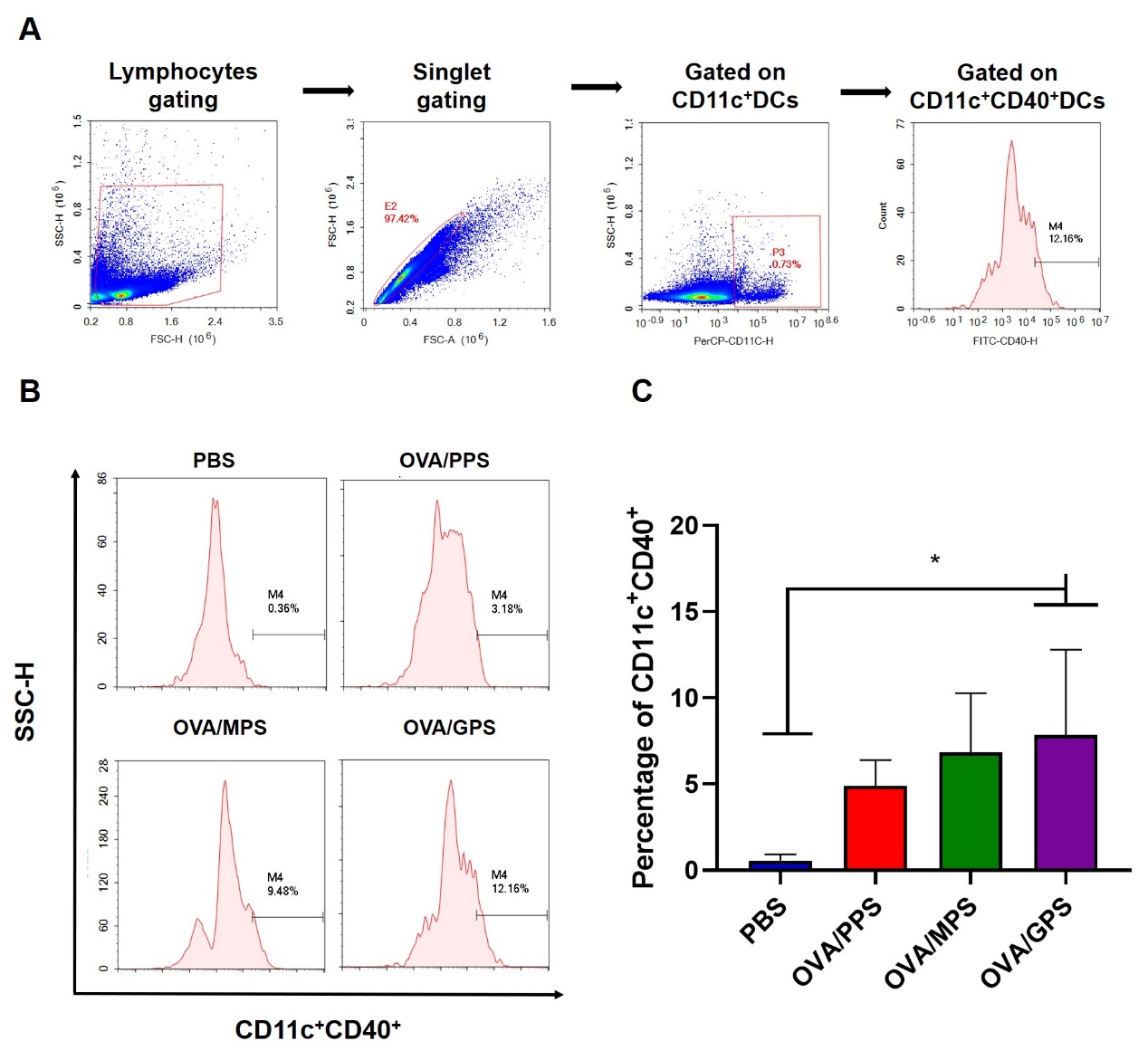


**Figure S5 OVA/GPS promote the activation of DCs in lymph nodes.** (A) The gating strategy and representative ﬂow cytometric quantification of activated DCs in lymph nodes after various treatments. (B) Representative flow cytometry dot plot showing CD40^+^DCs in lymph node 48 h after subcutaneous injection. (C) Statistics analysis of CD40^+^DCs in lymph node. The results are shown as mean ± SD (*n*=3), **p* < 0.5.


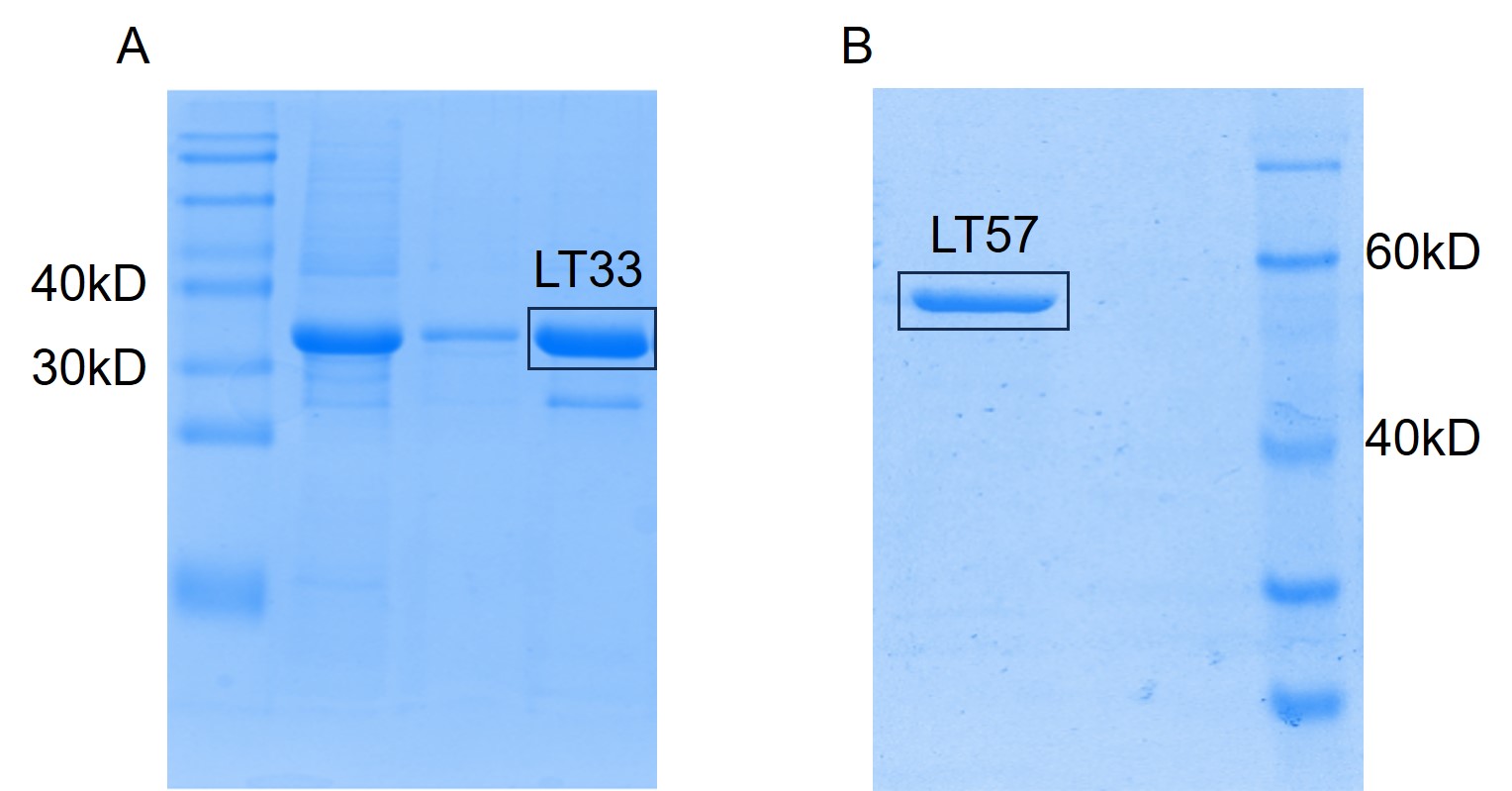


**Figure S6 The purified LT33 and LT57.** (A) Purification of LT33 verified with polyacrylamide gel electrophoresis. (B) Purification of LT57 verified with polyacrylamide gel electrophoresis.


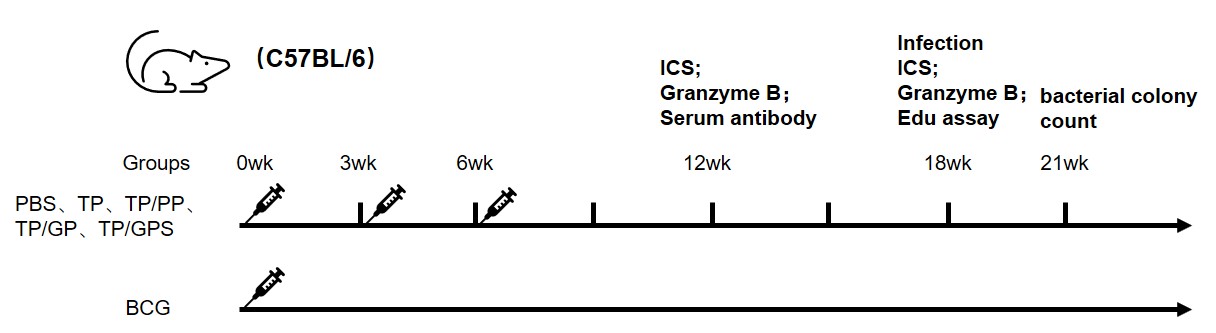


**Figure S7 vaccine immunization schedule.** The mice were divided into six groups: TP, TP/PP, TP/GP, TP/GPS, BCG, and PBS control. The BCG group received a single injection at week 0, while the other groups were immunized at weeks 0, 3, and 6. Cellular immune responses were evaluated 6 weeks after the last immunization. Immune memory was assessed 12 weeks after the last immunization, and protective efficacy was evaluated through an intranasal challenge with H37Ra (5 × 10^6^ CFU per mouse) at the same time point.


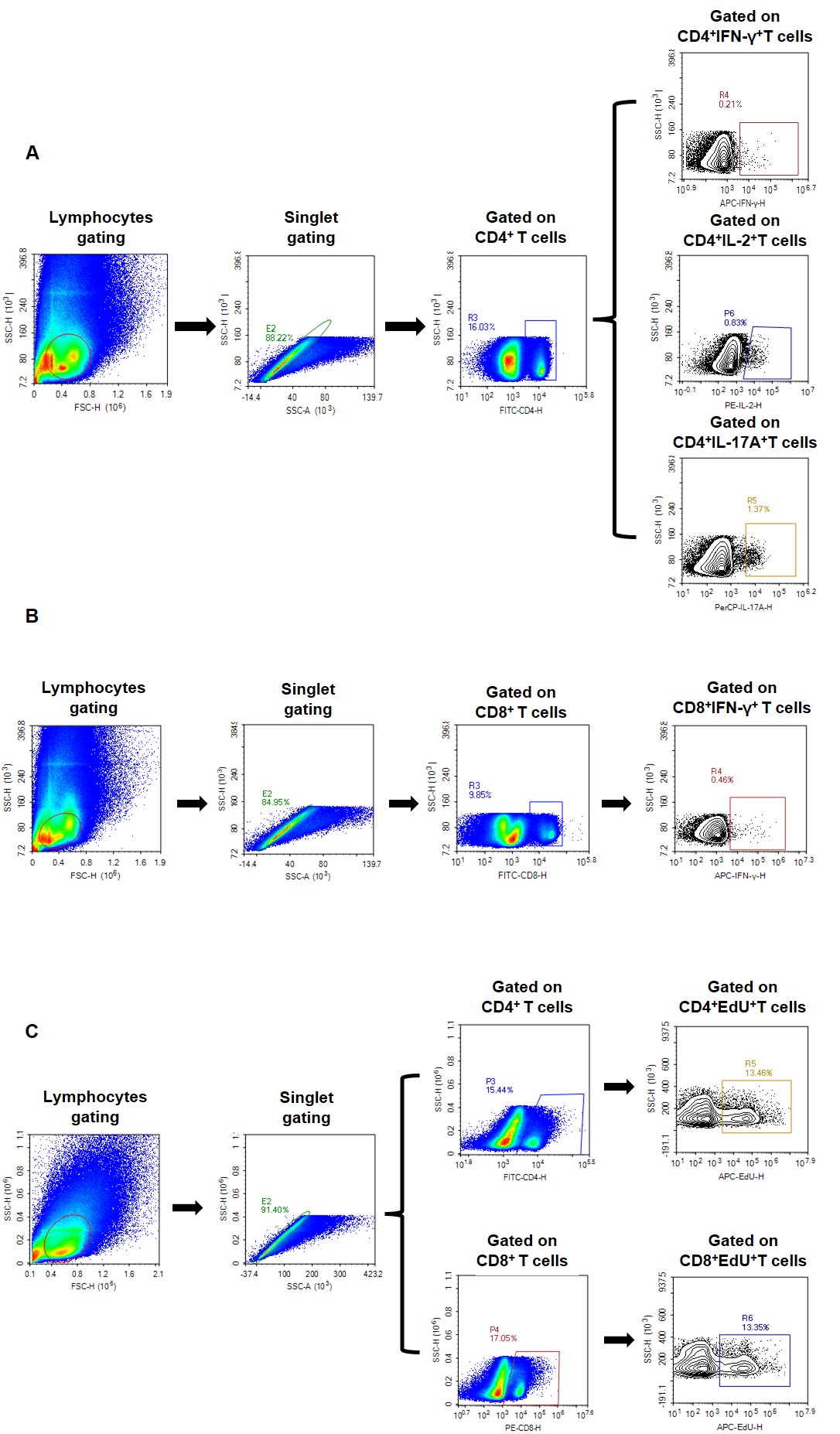


**Figure S8 Flow cytometry gating strategy.** (A) In intracellular cytokine staining assay, spleen lymphocytes were stained with the anti-CD4-FITC, anti-IFN-γ-APC, anti-IL-2-PE, and anti-IL-17A-PerCP-Cy5.5. Lymphocytes were first gated by the parameters SSC-H and FSC-H (lymphocytes), and then single cells were gated by the parameters FSC-H and FSC-A (single cells). Finally, CD4^+^IFN-γ^+^T cells, CD4^+^ IL-2^+^T cells, and CD4^+^ IL-17A^+^T cells were analyzed by flow cytometric. (B)Spleen lymphocytes were stained with the anti-CD8-FITC and anti-IFN-γ-APC. Lymphocytes were first gated by the parameters SSC-H and FSC-H (lymphocytes), and then single cells were gated by the parameters FSC-H and FSC-A (single cells). Finally, CD8^+^IFN-γ^+^T cells were analyzed by flow cytometric. (C) In EdU assays, spleen lymphocytes were stained with the anti-CD4-FITC and anti-CD8-PE. Lymphocytes were first gated by the parameters SSC-H and FSC-H (lymphocytes), and then single cells were gated by the parameters FSC-H and FSC-A (single cells). Finally, the CD4^+^EdU^+^T cells and CD8^+^EDU^+^T cells were analyzed by flow cytometric.
